# Macrophages upregulate mural cell-like markers and support healing of ischemic injury by adopting functions important for vascular support

**DOI:** 10.1038/s44161-024-00478-0

**Published:** 2024-06-06

**Authors:** Catarina Amoedo-Leite, Kristel Parv, Chiara Testini, Carmen Herrera-Hidalgo, Feifei Xu, Antoine Giraud, Marta Malaquias, Erik Fasterius, Daniel Holl, Cedric Seignez, Christian Göritz, Gustaf Christoffersson, Mia Phillipson

**Affiliations:** 1 Department of Medical Cell Biology, Uppsala University, Uppsala, Sweden; 2 National Bioinformatics Infrastructure Sweden, Science for Life Laboratory, Stockholm University, Solna, Sweden; 3 Department of Cell and Molecular Biology, Karolinska Institutet, Stockholm, Sweden; 4 Center for Neuromusculoskeletal Restorative Medicine, Hong Kong Science Park, Shatin, Hong Kong; 5 Science for Life Laboratory, Uppsala University, Uppsala, Sweden

**Keywords:** Monocytes and macrophages, Peripheral vascular disease

## Abstract

Sterile inflammation after injury is important for tissue restoration. In injured human and mouse tissues, macrophages were recently found to accumulate perivascularly. This study investigates if macrophages adopt a mural cell phenotype important for restoration after ischemic injury. Single-cell RNA sequencing of fate-mapped macrophages from ischemic mouse muscles demonstrates a macrophage-toward-mural cell switch of a subpopulation of macrophages with downregulated myeloid cell genes and upregulated mural cell genes, including *PDGFRβ*. This observation was further strengthened when including unspliced transcripts in the analysis. The macrophage switch was proven functionally relevant, as induction of macrophage-specific PDGFRβ deficiency prevented their perivascular macrophage phenotype, impaired vessel maturation and increased vessel leakiness, which ultimately reduced limb function. In conclusion, macrophages in adult ischemic tissue were demonstrated to undergo a cellular program to morphologically, transcriptomically and functionally resemble mural cells while weakening their macrophage identity. The macrophage-to-mural cell-like phenotypic switch is crucial for restoring tissue function and warrants further exploration as a potential target for immunotherapies to enhance healing.

## Abstract (web summary)

Amoedo-Leite et al. report that, in ischemic tissue, a subset of macrophages adopts mural cell-like morphology, gene expression and function, which is crucial for injury healing.

## Main

The unique properties of macrophages include their ability to traffic to and accumulate at distinct locations upon infection or tissue injury to exert their effector functions. In injured, ischemic tissues, these effector functions encompass clearing the site from dead and damaged cells^1^ as well as contributing to the formation of new vessels by paracrine release of growth factors and enzymes^2–5^. The first cells to arrive to the injured tissues are neutrophils, monocytes and macrophages, where the short-lived neutrophils are most numerous during the first couple of days after insult, whereas macrophages remain at the site and polarize into different subsets with distinct functions in response to environmental instructions^6,7^. Different macrophage subsets are, therefore, thought to contribute to restoration of tissue perfusion by separate means, for example by clearing the site of injured cells, as well as by producing distinct growth factors to promote angiogenesis and vessel stabilization^8,9^. To date, the wide range of known macrophage functions are attributed to their ability to switch into subsets within their lineage. Whether macrophages of adult tissues can adopt functions of other cells by initiating transdifferentiation has not been demonstrated.

Manifestations of cardiovascular diseases are caused by impaired tissue perfusion and subsequent injury and loss of tissue function. Rapid re-establishment of functional blood flow is critical after an ischemic event to limit the extent and severity of tissue damage as well as to allow for healing and regaining function. Formation of new blood vessels from existing ones, known as angiogenesis, is promptly initiated after ischemia onset and occurs in parallel with an inflammatory response provoked by the damaged tissue^9^. Angiogenesis is a highly complex process that involves degradation of the capillary basement membrane, endothelial cell proliferation and directed migration, followed by tube formation and vessel fusion^10^. Macrophages contribute to angiogenesis by producing vascular endothelial growth factor A (VEGF-A) and by degrading extracellular matrix (ECM)^9,11^. To regain functional perfusion, these vessels then need to stabilize and mature, which requires recruitment of perivascular mural cells (pericytes and smooth muscle cells) in a platelet-derived growth factor BB (PDGF-BB)-dependent (secreted by endothelial cells) and a platelet-derived growth factor receptor beta (PDGFRβ)-dependent (on the surface of mural cells) manner^12,13^. In a model of renal injury, macrophages were shown to support the recruitment of pericytes by secreting PDGF-BB^14^. During homeostasis, pericytes and vascular smooth muscle cells regulate vessel permeability and blood flow, respectively, and genetic models of mural cell loss result in hemorrhagic and dilated vessels, leading to lethality before birth^13,15^. So far, therapeutic means to improve the formation of functional blood vessels by upregulating growth factors or chemokines at the site of ischemic injury have shown limited clinical success^1,4,5,9^.

Using patient samples of the injured muscle after myocardial infarction and peripheral arterial disease, as well as ischemic mouse muscles, we recently found that macrophages accumulate at perivascular locations^11^. In the current study, we investigated if the perivascular macrophages in the ischemic muscle acquire a mural cell phenotype and gene expression profile important for re-establishing perfusion. Using two different mouse models of tissue ischemia, in combination with macrophage fate mapping, intravital microscopy and single-cell RNA sequencing (scRNA-seq) analyses, this study demonstrates that macrophages of adult mice can undergo a program to morphologically, transcriptomically and functionally resemble mural cells while downgrading their macrophage identity. The macrophages thereby shift toward a mural cell profile and undertake several mural cell functions important for healing of ischemic injury. This study uncovers an until now unknown role for perivascular macrophages in injured tissue, which may provide a potential target to promote the formation of functional blood vessels in ischemic disease.

## Results

### Macrophages adopt mural cell characteristics in injured muscle

**Fig. 1:**
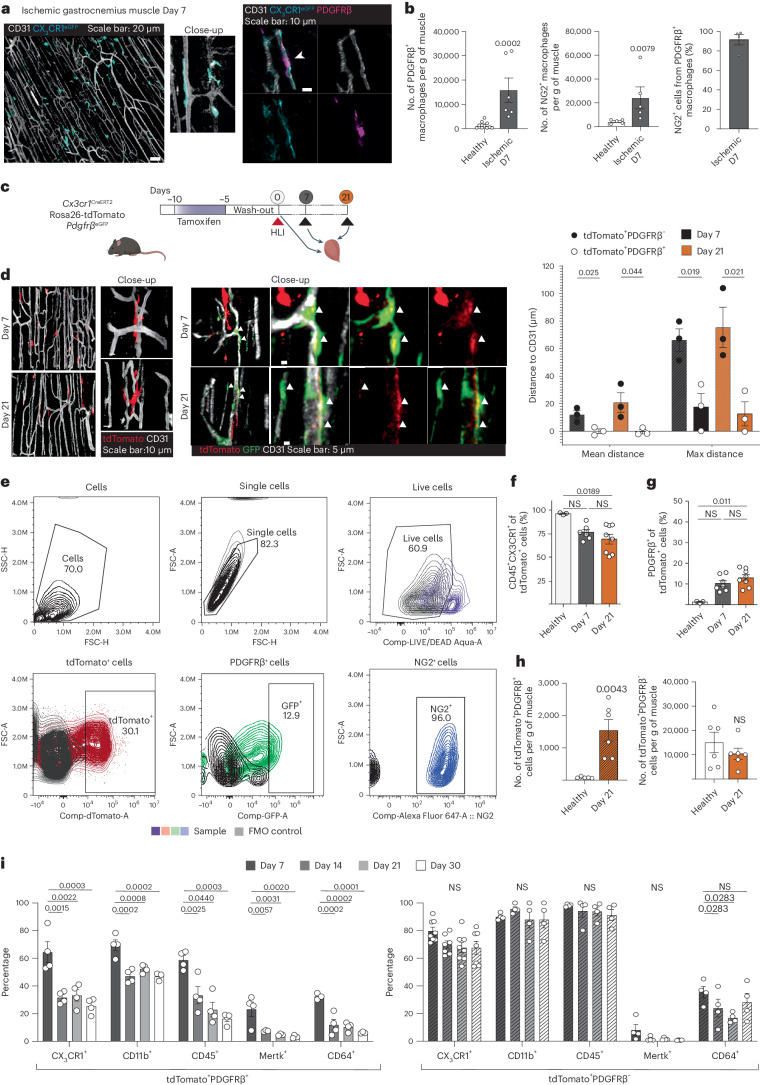
Macrophages accumulating in the ischemic muscle adopt a mural cell-like phenotype. **a**, Overview and close-up representative confocal whole-mount images of CX_3_CR1^+^ macrophages (cyan) in the ischemic hindlimb (day 7) demonstrate a mural cell-like morphology and localization near CD31^+^ vasculature (white). In ischemic muscles from *Cx3cr1*^*GFP/*+^ mice, perivascular CX_3_CR1^+^ macrophages (cyan, anti-GFP antibodies) are PDGFRβ^+^ (magenta, anti-PDGFRβ antibodies). **b**, At day 7 after ischemia, increased number of macrophages expressing PDGFRβ and NG2 mural cell markers were detected in ischemic muscles of *Pdgfrβ*^*eGFP*^ (*n* = 10(Healthy)-6(Ischemic Day 7)) and *Ng2*^*dsRed*^ (*n* = 5) reporter mice, respectively. **c**, Schematic representation of the lineage tracing strategy using the *Cx3cr1*^*CreERT2*^ *Rosa26*-tdTomato *Pdgfrβ*^*eGFP*^ mouse model where heritable tdTomato labeling of CX_3_CR1-expressing macrophages is induced by tamoxifen treatment. **d**, Representative overview and close-up images of tdTomato^+^ lineage-traced macrophages (red) and Pdgfrβ^eGFP+^ (green) cells at day 7 and day 21 after ischemia induction demonstrate tdTomato^+^Pdgfrβ^eGFP+^ mural cell-like macrophages in the *Cx3cr1*^*CreERT2*^ *Rosa26*-tdTomato *Pdgfrβ*^*eGFP*^ lineage-traced mouse muscles. Quantifications of the distances between tdTomato^+^Pdgfrβ^eGFP^^−^ or tdTomato^+^Pdgfrβ^eGFP^^+^ cell and CD31^+^ vasculature revealed that the tdTomato^+^Pdgfrβ^eGFP^^+^ cells were located much closer to the vasculature (*n* = 3). **e**, The gating strategy for tdTomato^+^, PDGFRβ^+^ and NG2^+^ cells in *Cx3cr1*^*CreERT2*^ *Rosa26*-tdTomato *Pdgfrβ*^*eGFP*^ lineage-traced cells. **f**, The percentage of tdTomato^+^ cells that express CD45 and CX_3_CR1 decreased in the ischemic muscle of *Cx3cr1*^*CreERT2*^ *Rosa26*-tdTomato *Pdgfrβ*^*eGFP*^ lineage-traced mice (*n* = 3(Healthy)-8(Ischemic Day 7 and 21)). **g**, The percentage of PDGFRβ-expressing tdTomato^+^ cells increased in the ischemic muscles of *Cx3cr1*^*CreERT2*^ *Rosa26*-tdTomato *Pdgfrβ*^*eGFP*^ lineage-traced mice (*n* = 3(Healthy)-8(Ischemic Day 7 and 21)). **h**, The number of tdTomato^+^ cells that express PDGFRβ (left) increased in the ischemic muscles, whereas the number of tdTomato^+^ cells that did not express PDGFRβ was the same at day 21 after ischemia onset as in healthy muscle (right) in the *Cx3cr1*^*CreERT2*^ *Rosa26*-tdTomato *Pdgfrβ*^*eGFP*^ lineage-traced mouse model (*n* = 6). **i**, Myeloid markers are reduced with time after ischemia in the tdTomato^+^PDGFRβ^+^ population (left) but not in the tdTomato^+^PDGFRβ^−^ cells (right) in the *Cx3cr1*^*CreERT2*^ *Rosa26*-tdTomato *Pdgfrβ*^*eGFP*^ lineage-traced mouse muscles (*n* = 4). Kruskal–Wallis followed by Dunn’s post hoc test (**f**,**g**). Two-tailed Mann–Whitney *U*-test (**b**,**h** (left)). Two-tailed unpaired Student’s *t*-test (**d**,**h** (right)). One-way ANOVA followed by Tukey test (**i**). Data are shown as average ± s.e.m. FSC-A, forward scatter area; FSC-H, forward scatter height; NS, not significant; SSC-H, side scatter height.

**Extended Data Fig. 1:**
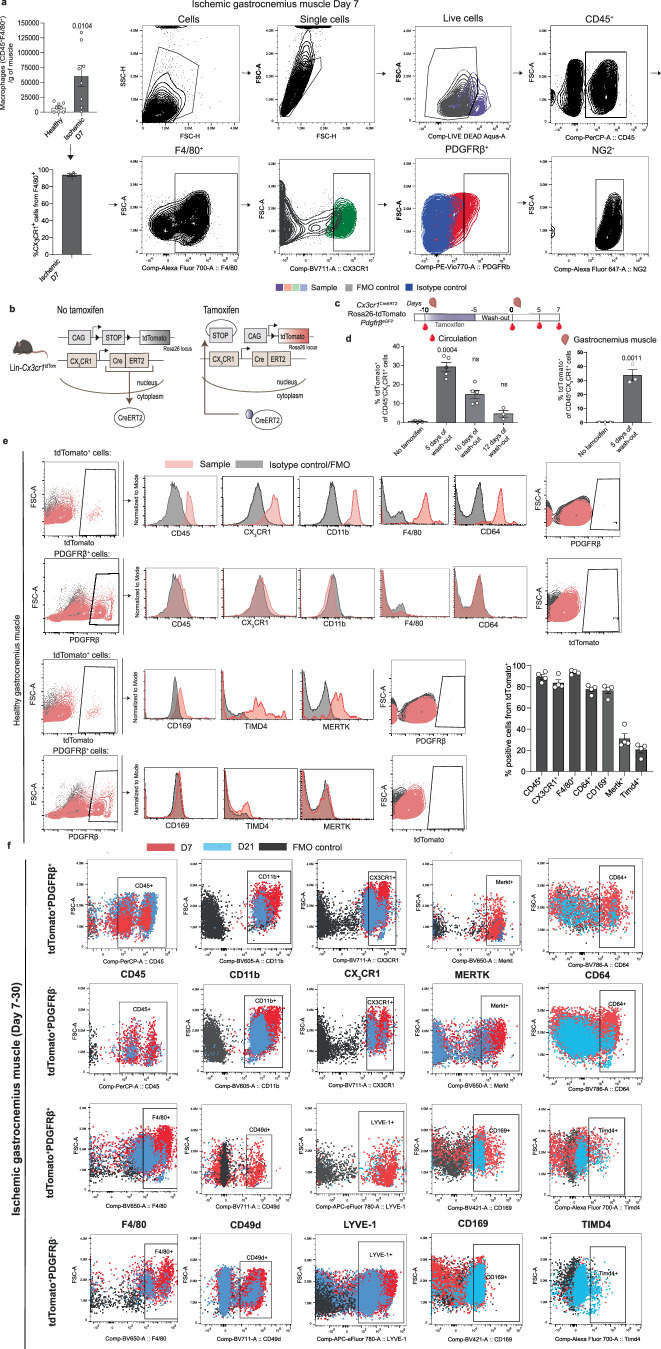
Muscle macrophages upregulate PDGFRβ upon ischemia. **a**) Number of CD45^+^ F4/80^+^ macrophages at day 7 after induction of ischemia in *Pdgfrβ*^*eGFP*^ and *Ng2*^*dsRed*^ reporter mice. Most CD45^+^ F4/80^+^ macrophages are CX_3_CR1^+^. Gating strategy on the right; n = 8. **b**) Schematic of the lineage tracing *Cx3cr1*^*CreERT2*^ *Rosa26*-TdTomato mice where tdTomato expression by macrophages is induced by tamoxifen treatment. **c**) Schematic representation of the experimental layout in the *Cx3cr1*^*CreERT2*^ *Rosa26*-tdTomato *Pdgfrβ*^*eGFP*^ lineage-traced mouse model. TdTomato expression is induced by tamoxifen treatment for 5 consecutive days and is followed by a 5-day wash-out period to remove tamoxifen from the system. **d**) Quantification of the tdTomato recombination efficiency of CD45^+^CX_3_CR1^+^ cells in blood according to the schematic in Extended Data Fig. 1c. n = 5 (Circulation)-3(Gastrocnemius muscle). **e**) Gating strategy and FMO controls of data from healthy muscle shown in Fig. 1f-h, and Extended Data Fig. 1d. In the graph, n = 4. Mann-Whitney U test (a); Kruskal-Wallis followed by Dunn’s post-hoc test, mean rank of each column was compared with the mean rank of “No tamoxifen” group (e). Data shown as average ± SEM.

Macrophages accumulated in the gastrocnemius muscle at day 7 after induction of hindlimb ischemia (HLI) (Extended Data Fig. 1a). Using whole-mount imaging and immunofluorescence, we found that macrophages were located preferentially at perivascular positions where they presented an elongated phenotype mimicking a mural cell-like morphology (Fig. 1a), as previously shown^11^, and that these macrophages expressed PDGFRβ (Fig. 1a). In fact, the number of macrophages that express the mural cell markers PDGFRβ and neural/glial antigen 2 (NG2) increased significantly at day 7 after ischemia, as determined by flow cytometry of single-cell suspensions from *Pdgfrβ*^eGFP^ and *Ng2*^*dsRed*^ reporter mice (Fig. 1b). In addition, 91.6 ± 5.4% of PDGFRβ^+^ macrophages also expressed NG2, as detected by surface staining (Fig. 1b; gating in Extended Data Fig. 1a). To decipher if this observation was due to macrophages upregulating mural cell markers or vice versa, we genetically lineage traced macrophages and studied their response to ischemic injury. For this, *Cx3cr1*^CreERT2^ mice were crossed with *Rosa26*-tdTomato mice, resulting in a heritable expression of tdTomato in myeloid cells including tissue-resident macrophages upon tamoxifen administration (Extended Data Fig. 1b). For simultaneous in situ detection of PDGFRβ expression, the *Cx3cr1*^CreERT2^ × *Rosa26*-tdTomato line was crossed with the *Pdgfrβ*^eGFP^ reporter line. Tamoxifen-mediated recombination was followed by a 5 day washout period before induction of HLI to ensure labeling specificity while taking the half-life of tamoxifen and its metabolites into consideration^16^ (Extended Data Fig. 1c). This approach resulted in 29.3 ± 2.4% and 33.7 ± 4.0% labeled macrophages in circulation and muscle, respectively, assessed at the time of ischemia induction (day 5 of washout; Extended Data Fig. 1d). Characterization of tdTomato^+^ cells in healthy muscles using flow cytometry (day 5 of washout) showed that the recombined cells are indeed macrophages (Extended Data Fig. 1e). Notably, we also found that PDGFRβ^+^ cells did not express leukocyte markers before ischemia induction (Extended Data Fig. 1e), validating our lineage tracing strategy.

**Fig. 2:**
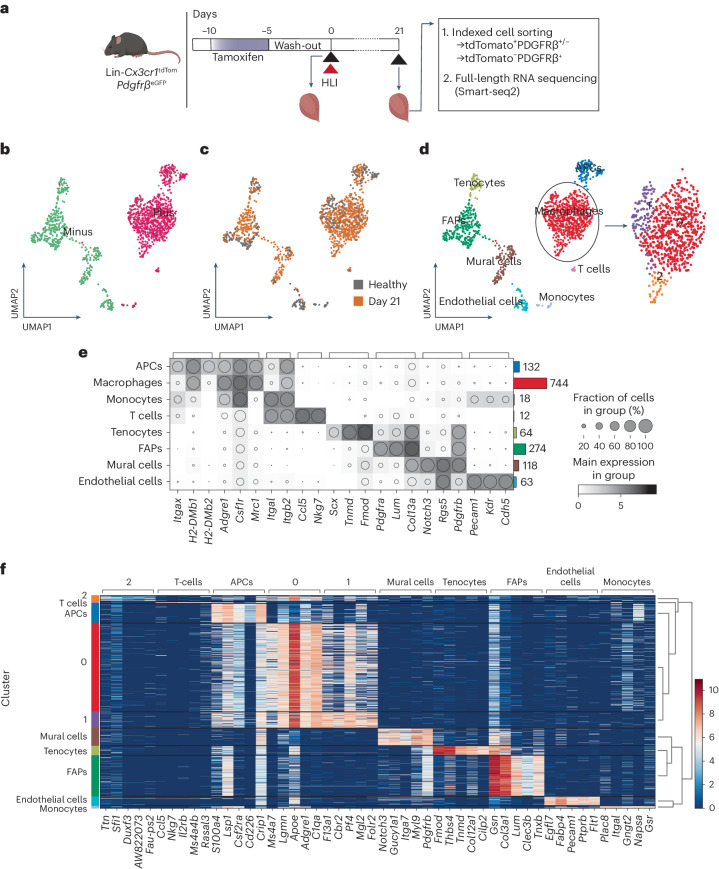
Single-cell RNA profiling of tdTomato^+^ and tdTomato-PDGFRβ^+^ cells in healthy and day 21 post-ischemic muscle. **a**, Experimental layout of the scRNA-seq experiment, where *Cx3cr1*^*CreERT2*^ *Rosa26*-tdTomato *Pdgfrβ*^*eGFP*^ lineage-traced mice received tamoxifen treatment for 5 days, followed by a 5 day washout period before induction of HLI. Single cells were isolated from healthy mice and at D21 after ischemia induction and thereafter sorted based on tdTomato and PDGFRβ-eGFP signal. UMAP embedding of scRNA-seq data showing the cells colored by tdTomato signal (**b**), timepoint (healthy or D21 after ischemia) (**c**) and assigned cell types with macrophage subclustering (**d**). **e**, Dot plot of known marker genes for the assigned cell types with the number of cells in each cell type in bar plot to the right. **f**, Heatmap showing the top five genes driving the separation of the cell type clusters and macrophage subclusters (*n* = 5).

**Extended Data Fig. 2:**
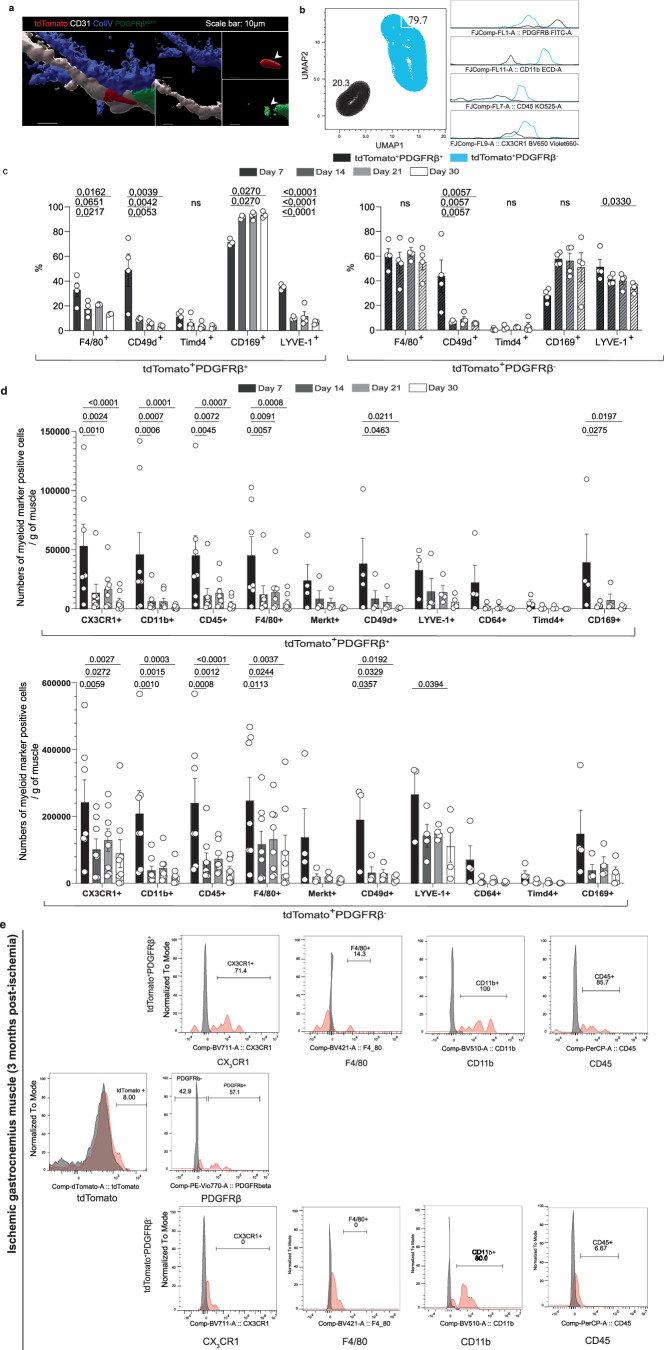
Muscle macrophages downregulate myeloid markers post-ischemia induction. **a**) Gating strategy and FMO controls of data from ischemic muscle shown in Fig. 1i and Extended Data Fig. 1J-K. **b**) Representative image of a PDGFRβ^+^ (green) tdTomato^+^ cell (red) localized between the collagen IV^+^ basement membrane (blue) and CD31^+^ vasculature (white) in the ischemic muscle of *Cx3cr1*^*CreERT2*^ *Rosa26*-tdTomato *Pdgfrβ*^*eGFP*^ lineage-traced mice. **c**) FlowJO UMAP plugin analysis reveals TdTomato^+^ lineage traced cells cluster into PDGFRβ^+^CD11b^low^CD45^low^CX_3_CR1^low^ (black) and PDGFRβ^-^CD11b^high^CD45^high^CX_3_CR1^high^ populations (blue). Cells isolated from day 21 ischemic muscle of *Cx3cr1*^*CreERT2*^ *Rosa26*-tdTomato *Pdgfrβ*^*eGFP*^ mice. **d**) Myeloid markers are reduced with time following ischemia in the tdTomato^+^PDGFRβ^+^ population (left) but not in the tdTomato^+^PDGFRβ^-^ cells (right) in the *Cx3cr1*^*CreERT2*^ *Rosa26*-tdTomato *Pdgfrβ*^*eGFP*^ lineage-traced mouse model. Graphs related to Fig. 1i. n = 4. **e**) Numbers of cells expressing myeloid markers following ischemia in the tdTomato^+^PDGFRβ^+^ population (upper) but not in the tdTomato^+^PDGFRβ^-^ cells (down) in the *Cx3cr1*^*CreERT2*^ *Rosa26*-tdTomato *Pdgfrβ*^*eGFP*^ lineage-traced mouse model. n = 4. K) Gating strategy and FMO controls of muscle from mice at 3 months post-ischemia induction. One-way ANOVA followed by Turkey test (d-e). Data shown as average ± SEM.

Consequently, we induced ischemia in mice with lineage-traced macrophages (see Fig. 1c for experimental outline) and found, through in vivo imaging, that tdTomato^+^ macrophages took on perivascular positions in the ischemic muscle (at day 7 and day 21 after ischemia induction) (Fig. 1d). Approximately 20% of the TdTomato^+^ cells also expressed GFP (day (D) 7: 20.51 ± 2.54%, D21: 17.78 ± 6.41%), and the tdTomato^+^PDGFRβ^+^ cells were found closer to the CD31^+^ blood vessels when compared to the tdTomato^+^PDGFRβ^−^ cells at both timepoints (Fig. 1d). Notably, flow cytometry of single-cell suspensions of muscles (gating strategy in Fig. 1e and Extended Data Fig. 1f) revealed that approximately 25–30% of tdTomato^+^ macrophages downregulated the expression of the myeloid markers CD45 and CX_3_CR1 (Fig. 1f), whereas 10.3 ± 1.4% and 13.0 ± 1.5% upregulated the mural cell marker PDGFRβ at D7 and D21 after HLI (Fig. 1g), suggesting a macrophage-to-mural cell adaptation in response to ischemia. More than 80% of the lineage-traced cells that expressed PDGFRβ after ischemia induction also expressed NG2 (Fig. 1e; D7: 88.2 ± 2.8%). Immunofluorescent evaluation of tissue sections demonstrated that, 3 weeks after HLI induction, tdTomato^+^ cells were PDGFRβ^+^ and were located within the collagen IV^+^ endothelial basement membrane (Extended Data Fig. 2a), a position shared with pericytes^12^. Interestingly, although the numbers of PDGFRβ^−^ lineage-traced macrophages were similar before ischemia and at later stages of ischemia onset, PDGFRβ^+^ lineage-traced macrophages accumulated in ischemic hindlimbs (Fig. 2b). Furthermore, uniform manifold approximation and projection (UMAP) analysis revealed that approximately 20% of the tdTomato^+^ cells expressed higher levels of PDGFRβ while simultaneously expressing lower levels of several macrophage-associated markers (Extended Data Fig. 2b). In fact, downregulation of several myeloid markers was specifically detected in PDGFRβ^+^ lineage-traced macrophages, where several of these were further reduced with time (Fig. 1i and Extended Data Fig. 1c,d). Very few lineage-traced macrophages could be detected at 3 months after ischemia induction (319.2 ± 95.6 cells per gram of muscle compared to >10,000 tdTomato^+^ cells per gram of muscle at D7–D30 after ischemia). Of these, the PDGFRβ^+^ cells still expressed some macrophage-associated proteins, whereas the PDGFRβ^−^ cells retained only their CD11b expression (Extended Data Fig. 2e). These data demonstrate that macrophages adopt mural cell-like characteristics during the healing phase of ischemic muscle injury.

### scRNA-seq reveals macrophages with a mural cell profile

**Extended Data Fig. 3:**
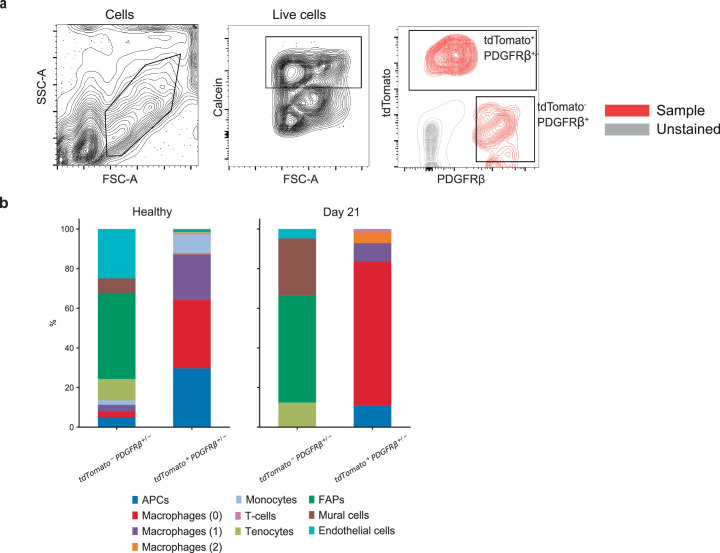
Cell populations of the healthy and ischemic muscle. **a**) Gating strategy for FACS-sorting of tdTomato^+^ PDGFRβ^+/-^ and tdTomato^−^PDGFRβ^+^ cells from healthy and ischemic muscle from *Cx3cr1*^*CreERT2*^ *Rosa26*-tdTomato *Pdgfrβ*^*eGFP*^ lineage-traced mice used for the scRNA-seq experiments. Live cells stained with Calcein. **b**) Cell type and macrophage sub-cluster percentage from the scRNA-seq data, demonstrating shifts in cell type composition between Healthy and Day 21 post-ischemia in both tdTomato^-^ PDGFRβ^+/-^ and tdTomato^+^PDGFRβ^+/-^ cells from *Cx3cr1*^*CreERT2*^ *Rosa26*-tdTomato *Pdgfrβ*^*eGFP*^ lineage-traced mice. n = 5.

To assess the extent of macrophage-to-mural cell shift after ischemia, we performed scRNA-seq of FACS-sorted tdTomato^+^ (predominantly of macrophage origin) and tdTomato^−^PDGFRβ^+^ (predominantly mural cells and fibroblasts) cells using healthy and ischemic (day 21 after ischemia) gastrocnemius muscle from lineage-traced *Cx3cr1*^CreERT2^ × *Rosa26*-tdTomato × *Pdgfrβ*^eGFP^ mice (Fig. 2a and Extended Data Fig. 3a) at two distinct timepoints.

**Extended Data Fig. 4:**
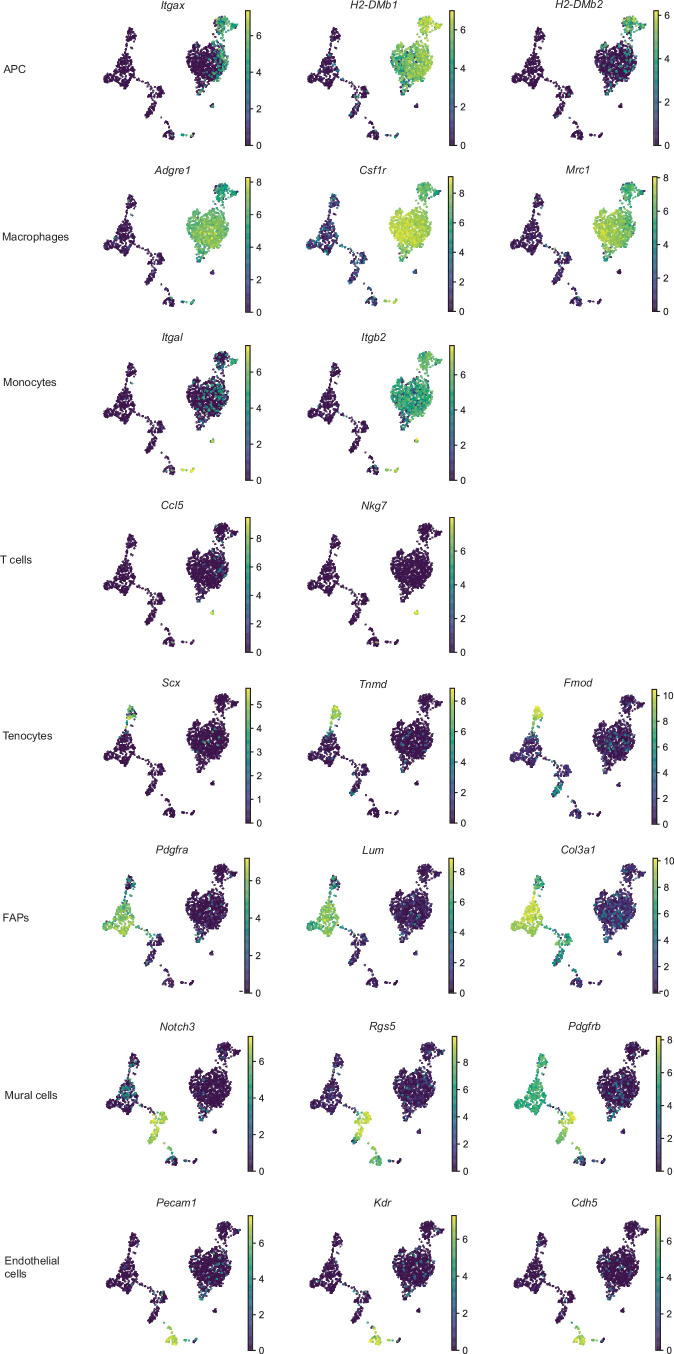
Gene expression of the different cell populations analyzed by single cell sequencing. Related to Fig. 2d, E and F, UMAP visualization of selected genes enriched in the cell type clusters identified in the scRNA-seq experiments. n = 5.

After integration of the batches, 1,425 cells passed the quality assessment, of which 148 and 737 were tdTomato^+^PDGFRβ^+^ from the healthy and ischemic muscles (day 21 after ischemia), respectively, whereas 177 and 363 were tdTomato^−^PDGFRβ^+^ (Fig. 2b,c). Antigen-presenting cells (APCs) (132 cells) and macrophages (744 cells, three subclusters 0, 1 and 2) formed distinct clusters among tdTomato^+^ cells, and mural cells (118 cells), fibro/adipogenic progenitors (FAPs) (274 cells), tenocytes (64 cells) and endothelial cells (63 cells) formed distinct clusters among tdTomato^−^ cells (Fig. 2d–f and Extended Data Figs. 3b and 4). Furthermore, 21 cells that were tdTomato^−^ were identified as macrophages or APCs (Fig. 2b,d), indicative of incomplete recombination efficiency with tamoxifen treatment, as previously shown in Extended Data Fig. 1e.

**Fig. 3:**
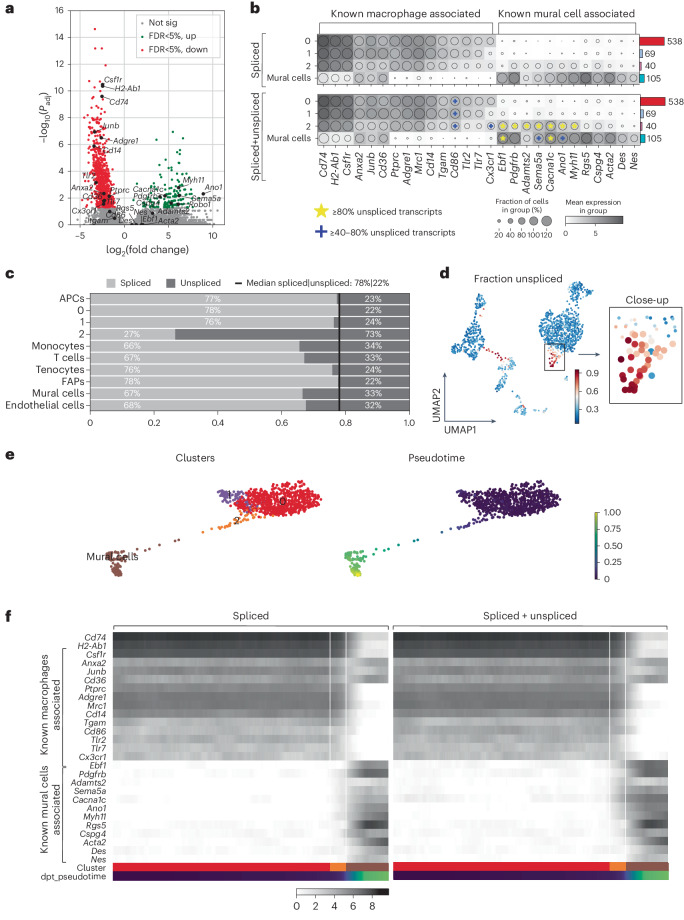
A subset of macrophages expresses mural cell-associated genes while expressing lower levels of immune cell-associated genes. **a**, Volcano plot of DEGs comparing cluster 2 to cluster 0. Each dot represents a gene; *x* axis represents the log_2_ fold change of gene expression in comparison; *y* axis represents the −log_10_-transformed adjusted *P* values. Statistical method, Wilcoxon rank-sum test; adjusted *P* value, Benjamini–Hochberg. Genes with FDR-adjusted *P* < 5% were considered to be significant, with higher and lower expression genes marked green and red, respectively. Non-significant genes are in gray. Some examples of mural cell-associated and macrophage-associated transcripts are highlighted. **b**, Dot plot of known macrophage-associated and mural cell-associated gene expressions without (Spliced) or with (Spliced+Unspliced) unspliced transcripts in different clusters, with ‘*’ marking genes with ≥80% unspliced transcript fraction and ‘+’ marking genes with ≥40–80% unspliced transcript fraction. **c**, Proportions of spliced and unspliced transcripts in all the clusters, with median value expressed as the black line, showing that cluster 2 has a high proportion of unspliced transcripts. **d**, Clustered cells colored by fraction of unspliced transcripts, with cluster 2 (close-up on the right) standing out as having a high proportion of unspliced transcripts. Big dots, cluster 2; small dots, clusters 0 and 1 (only in close-up). **e**, Trajectory inference analysis by PAGA of macrophage subclusters and mural cells. Clusters (left) and pseudotime (right). **f**, Expression of known macrophage-associated and mural cell-associated genes in pseudotemporally ordered cells (left: spliced transcript only, right: spliced and unspliced transcripts combined). All the analyses performed are based on D21 post-ischemia cells isolated from the *Cx3cr1*^*CreERT2*^ *Rosa26*-tdTomato *Pdgfrβ*^eGFP^ lineage-traced mouse model (*n* = 5). Not sig, not significant.

**Extended Data Fig. 5:**
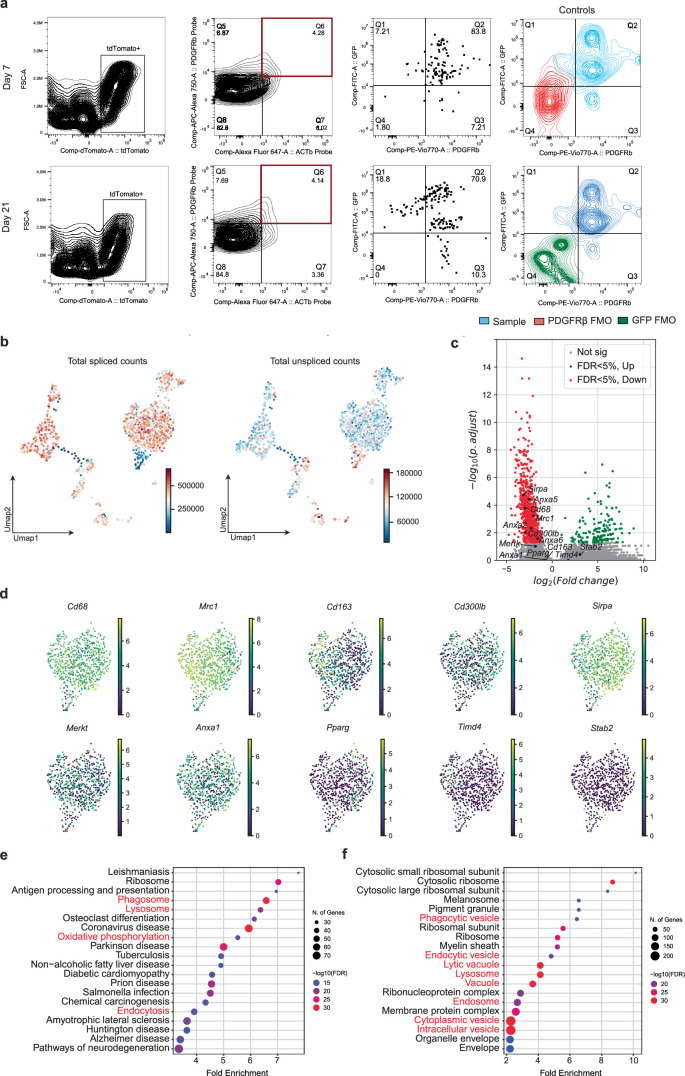
Gene expression comparisons between macrophage cluster 2 and 0. **a**) Primeflow experiment results showing the gating of tdTomato^+^ cells, followed by selection of the cells that were positive for both the *PDGFRβ*-RNA probe and for the housekeeping gene *ACTβ*-RNA probe (red square/Q6). At days 7 and 21 post-ischemia, the majority of cells expressing *PDGFRβ*-RNA are positive for PDGFRβ surface protein and GFP (Q1). **b**) Relative to Fig. 3d, UMAP embedding of scRNA-seq data colored by numbers of total spliced and unspliced counts. **c**) Volcano plot of differentially expressed genes comparing cluster 2 to 0. Each dot represents a gene, x-axis represents the log2 fold change of gene expression in comparison, y-axis represents the -log10-transformed adjusted p-values. Genes with False Discovery Rate (FDR) adjusted p-value < 5% were considered to be significant, with genes with higher and lower expression marked green and red, respectively. Non-significant genes in grey. Phagocytosis-associated transcripts are highlighted. **d**) UMAP visualization of selected phagocytosis-associated transcripts in the macrophage cluster. **e**) Top 20 significant KEGG and **f**) Cellular Component GO terms enriched in the transcripts with significantly lower expression in cluster 2 compared to cluster 0. Phagocytosis-associated pathways are marked in red. n = 5.

Macrophages of healthy muscle predominantly belonged to macrophage cluster 1, whereas most macrophages of ischemic muscle were found in cluster 0, indicating an ischemia-induced expression signature (Fig. 2c–e and Extended Data Figs. 3b and 4). Interestingly, macrophage cluster 2 appeared only in the ischemic muscle, suggesting that these macrophages emerged after ischemia induction. The top genes driving the separation of the clusters in the ischemic muscle are presented in Fig. 2f (Supplementary Table 1). Among the differentially expressed genes (DEGs) between clusters 2 and 0 in ischemic muscle, 168 genes were expressed to a higher degree, whereas 1,068 genes were expressed at lower levels in cluster 2 at 3 weeks after ischemic injury (Fig. 3a and Supplementary Table 2). Of these, the genes expressed significantly more in cluster 2 were associated with mural cells, whereas macrophage-associated genes were expressed at significantly lower levels (Fig. 3a,b). Cluster 2 was demonstrated to correspond to the mural cell-like macrophages observed in Fig. 1, as the *Pdgfrb* mRNA levels showed high correlation with both GFP and PDGFRβ protein expression (Extended Data Fig. 5a). Adding unspliced transcripts on top of the spliced transcripts, it became visually more apparent that the highly expressed genes in cluster 2 associated with mural cells (*Pdgfrb*, *Adamts2*, *Ano1*, *Sema5a*, *Cacna1c* and *Myh11*, ‘*’ in Fig. 3b, which all have a high fraction of unspliced transcripts), whereas macrophage-associated genes generally had a low fraction of unspliced transcripts (with the exception of *Cd86*, ‘+’ in Fig. 3b), reflecting an ongoing induction of gene expression and, therefore, the future state of the cells^17^. Cluster 2 contained a high proportion of unspliced transcripts at 73% compared to an average of 26% unspliced sequences (Fig. 3c), and the average unspliced level is in agreement with the general observation in scRNA-seq data^17^. This high fraction of unspliced transcripts was also maintained at cellular level (Fig. 3d and Extended Data Fig. 5b).

Genes highly expressed in cluster 2 compared to cluster 0 were also highly expressed in mural cells (Fig. 3a,b). *Ano1*, also known as *Tmem16a*, is a calcium-activated chloride channel previously demonstrated to be required for peripheral blood vessel contractility causing disturbances in blood pressure after gene inactivation in mice^18^. *Myh11* is known to be expressed in mature smooth muscle cells and pericytes to generate smooth muscle contractile protein myosin heavy chain 11 (myosin-11)^19^. Other mural cell-associated genes, including *Pdgfrb*, *Sema5a*, *Adamts2* and *Cacna1c*, were also highly expressed in cluster 2 (ref. ^20^) (Fig. 3a,b) in agreement with our observations of elevated protein levels of PDGFRβ in ischemic muscles (Fig. 1f). Expression of other mural cell markers, including *Rgs5*, *Nes*, *Acta2* and *Des*, were nevertheless not expressed significantly more in cluster 2 (Fig. 3a,b)^20^. Furthermore, *Ebf1*, encoding early B cell factor-1 transcription factor, was not among the genes with increased expression in cluster 2 (Fig. 3a) but had a high fraction of the unspliced transcripts (Fig. 3b). EBF1-expressing perivascular cells have been suggested to represent pericytes, and EBF1 has a functional role in the cell fate commitment toward the pericytes phenotype^21^. Regarding genes associated with immune cell functions, *Ptprc* and *Cx3cr1* were both expressed at lower levels in cluster 2 when compared to macrophage cluster 0 (Fig. 3a), again confirming our observations at the protein level (Fig. 1i). In addition, significantly lower expression levels of various hallmark innate immunity genes were observed in cluster 2, including toll-like receptors (*Tlr2* and *Tlr7*), which play key roles in the detection of pathogens and consecutive macrophage activation. Compared to the macrophage cluster 0, the macrophages of cluster 2 expressed significantly lower levels of transcripts associated with phagocytosis (*CD68*, *Mrc1*, *CD300lb*, *Sirpa*, *Anxa2*, *Anxa5* and *Anxa6*; Extended Data Fig. 5c,d). This indicates poor phagocytic ability for the cells within cluster 2, which was also highlighted by the top 20 significant KEGG categories and the Gene Ontology (GO) cell component categories of genes that were significantly reduced in cluster 2 compared to cluster 0 (Extended Data Fig. 5e,f).

**Fig. 4:**
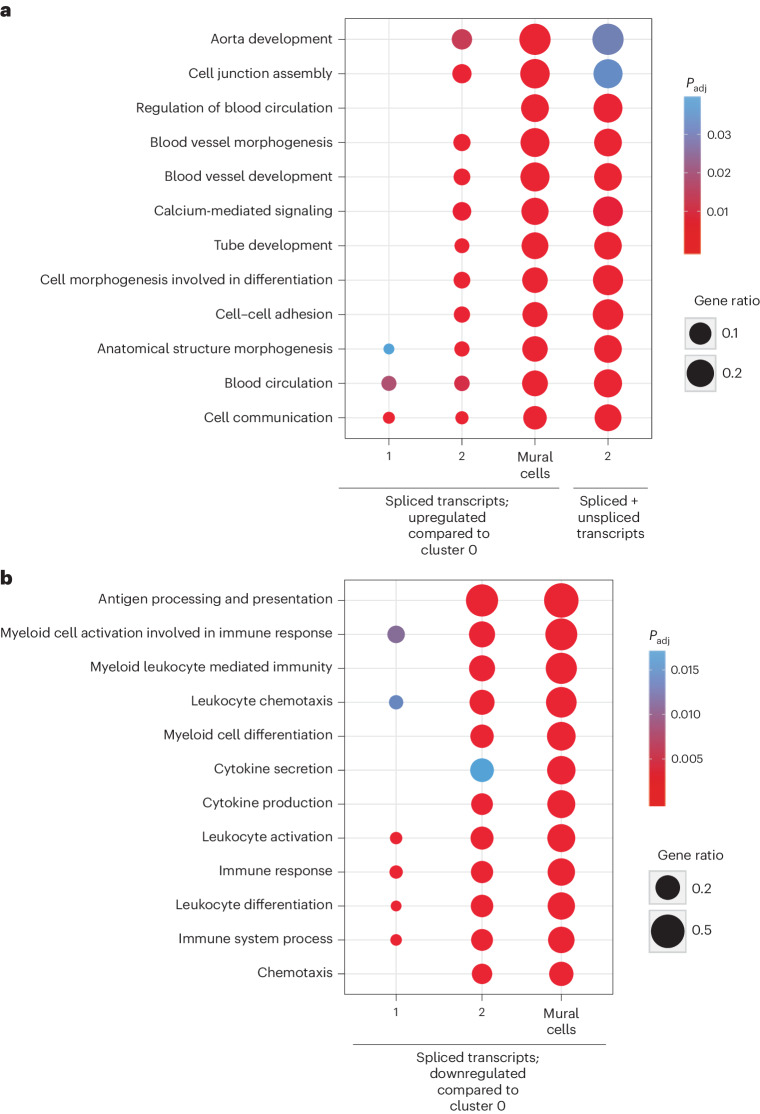
Enrichment of GO terms associated with mural cell functions and depletion of those associated with immune cell function in cluster 2. Enrichment (**a**) and depletion (**b**) of GO terms in tdTomato^+^ macrophage subclusters 1 and 2 and PDGFRβ^+^ clusters of mural cells, compared to cluster 0, with transcripts of high unspliced fraction in cluster 2 (only in **a**). GO terms were selected based on the overlap between cluster 2 and mural cells (73.5%) and cluster 2 and macrophages from cluster 0 (72.7%) (*n* = 5). FDR (*P*_adj_) was calculated using the Benjamini–Hochberg method to control for multiple hypothesis testing from the gene enrichment analysis performed with STRING version 11.0b.

**Extended Data Fig. 6:**
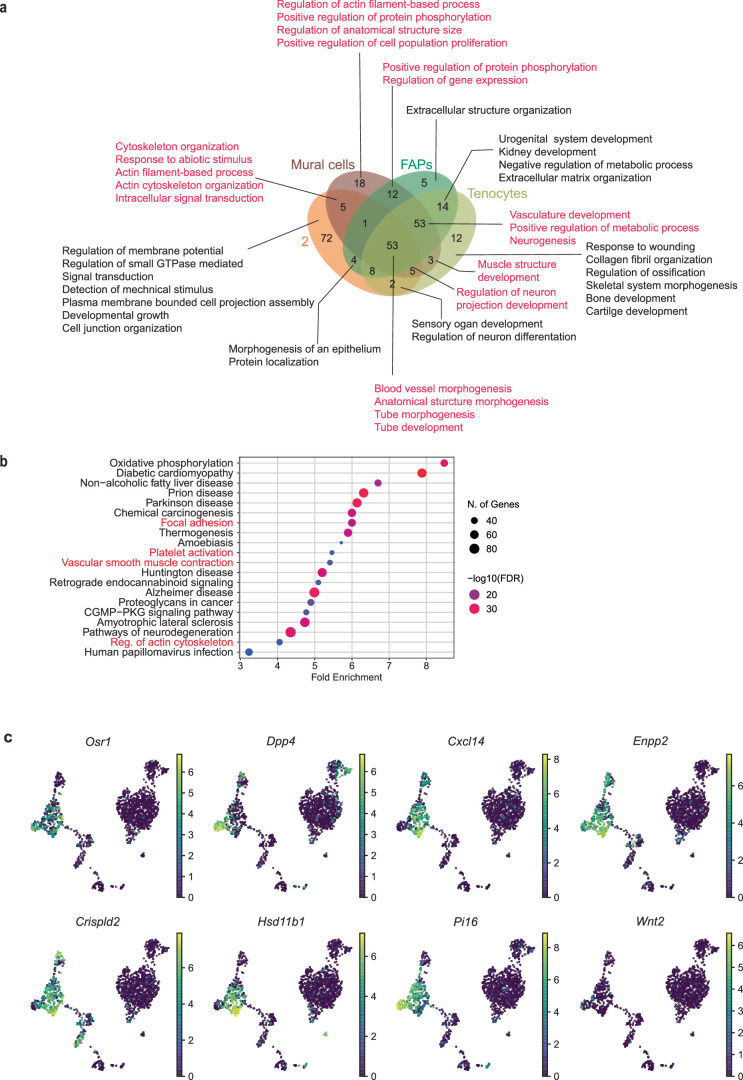
Gene expression comparisons between macrophage cluster 2, tenocytes, FAPs and mural cells. **a**) Venn Diagram of Top 150 GO terms enriched in clusters 2, tenocytes, FAPs and mural cells when comparing to cluster 0, with a selection of pathways shown. Pathways identified as enriched in mural cell cluster are marked red. **b**) Top 20 significant KEGG enriched in the transcripts with significantly lower expression in cluster 2 compared to cluster 8. Pathways associated with mural cell function are marked in red. **c**) UMAP plots with color-coded expression of the genes characteristic of FAPs as described by Oprescu et al., 2020. n = 5.

Trajectory inference revealed the pseudotemporal ordering of the ischemic muscle cells, which identified cluster 2 as an ‘intermediate’ population between cluster 0 macrophages and mural cells (Fig. 3e). Analysis of known macrophage and mural cell markers along this transdifferentiating path reveals a decreasing expression of macrophage-associated markers and increasing expression of various mural cell markers discussed above (for example, *Pdgfrb*, *Ano1* and *Myh11*) (Fig. 3f). Inclusion of unspliced transcripts in this analysis further highlighted the increased expression of mural cell markers (Fig. 3f). Gene set enrichment analysis performed using STRING^22^ with genes expressed to a higher degree in cluster 2 (versus cluster 0) yielded various GO biological process terms (referred to as GO terms) associated with mural cell functions^23^ in the ischemic muscle (Fig. 4a, Extended Data Fig. 6a and Supplementary Table 3), including blood circulation, blood vessel development, cell junction assembly, anatomical structure morphogenesis and tube development. In total, 73.5% of GO terms enriched in cluster 2 overlapped with those enriched in mural cell clusters. In contrast, of the GO terms depleted in cluster 2, 72.7% were associated with those enriched in cluster 0 macrophages, including immune system process, positive regulation of immune system process, immune response, myeloid cell activation in immune response, cytokine secretion and leukocyte differentiation (Fig. 4b). Interestingly, genes significantly expressed to a lower degree in cluster 2 versus in the mural cell cluster unveiled KEGG pathways associated with certain mural cell functions, including vascular smooth muscle contractility, suggesting that not all mural cell characteristics are acquired by cluster 2 macrophages (Extended Data Fig. 6b and Supplementary Table 3). Various overlapping terms between cluster 2 and those of tenocytes (tendon fibroblasts) and FAPs, previously shown to be related to mural cells and the production and remodeling of ECM, were also revealed (Extended Data Fig. 6a,c)^24,25^. Genes with high unspliced numbers of transcripts from cluster 2 were also enriched in mural cell-associated biological processes (68.9%; Fig. 4a), indicating an ongoing expression shift of cluster 2 toward a mural cell identity.

In summary, the scRNA-seq data demonstrate that, 3 weeks after ischemia induction, a subpopulation of muscle macrophages acquires a gene expression profile characteristic of mural cells while simultaneously downregulating the expression profile associated with macrophages.

### Macrophages take on mural cell position and functions

**Extended Data Fig. 7:**
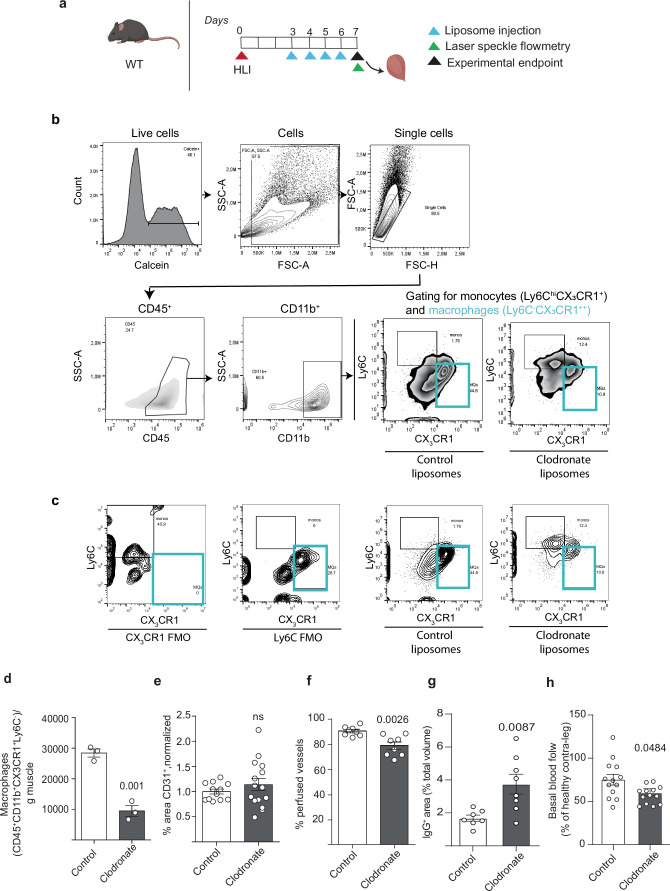
Macrophages in the ischemic muscle adopt mural cell functions. **a**) Schematic representation of the experimental layout. **b**) Gating strategy for macrophages in macrophage depletion experiments in C57Bl/6 J mice. **c**) FMO controls for Ly6C and CX_3_CR1 flow cytometry staining (related to Extended Data Fig. 6b). **d**) The numbers of CD45^+^CD11b^+^CX3Cr1^+^Ly6C^−^ macrophages were reduced in the clodronate liposome treated ischemic muscles from C57Bl/6 J mice, where number of macrophages were normalized by gram of muscle. **e**) The CD31 positive area was unaffected in clodronate liposome treated ischemic muscles from C57Bl/6 J mice, expressed as percentage to average of control liposomes treated group. **f**) The percentage of perfused vessels were reduced in clodronate liposome treated ischemic muscles from C57Bl/6 J mice, quantified as double stained with lectin administered *in vivo* and CD31 stained *ex vivo* and expressed as percentage of CD31^+^ vessels. **g**) Vessel leakage was increased in clodronate liposome treated ischemic muscles from C57Bl/6 J mice, measured as extravascular IgG staining and shown as percentage of the total image volume. **h**) Basal perfusion was reduced in ischemic muscles of clodronate liposome treated C57Bl/6 J mice, measured as percentage of basal perfusion of the healthy contra-leg in the same mouse. All the analysis performed here are from Day 7 post-ischemia muscles; n = 3. Two^−^tailed unpaired student’s t-test (d, f-h) and two^−^tailed Mann-Whitney U test (e). Data shown as average ± SEM.

Next, we asked if the macrophages were necessary for vessel maturation. The macrophages were depleted before downgrading their macrophage characteristics and at a timepoint where the ischemia-induced angiogenesis is already initiated, as macrophages are known to also induce angiogenesis, by administrating clodronate liposomes daily between days 3 and 7 after ischemia induction. This approach resulted in a 67 ± 8% depletion of macrophages in ischemic muscles (see Extended Data Fig. 7a–c for gating strategy and Extended Data Fig. 7d for verification of depletion), whereas vessel density was not affected (day 7 after ischemia; Extended Data Fig. 7e). Furthermore, macrophage depletion decreased the number of perfused vessels (shown as reduced lectin:CD31 ratio; Extended Data Fig. 6f), increased vessel permeability (Extended Data Fig. 7g) and reduced basal tissue perfusion (Extended Data Fig. 7h), demonstrating a role of macrophages in vessel maturation after ischemia.

**Fig. 5:**
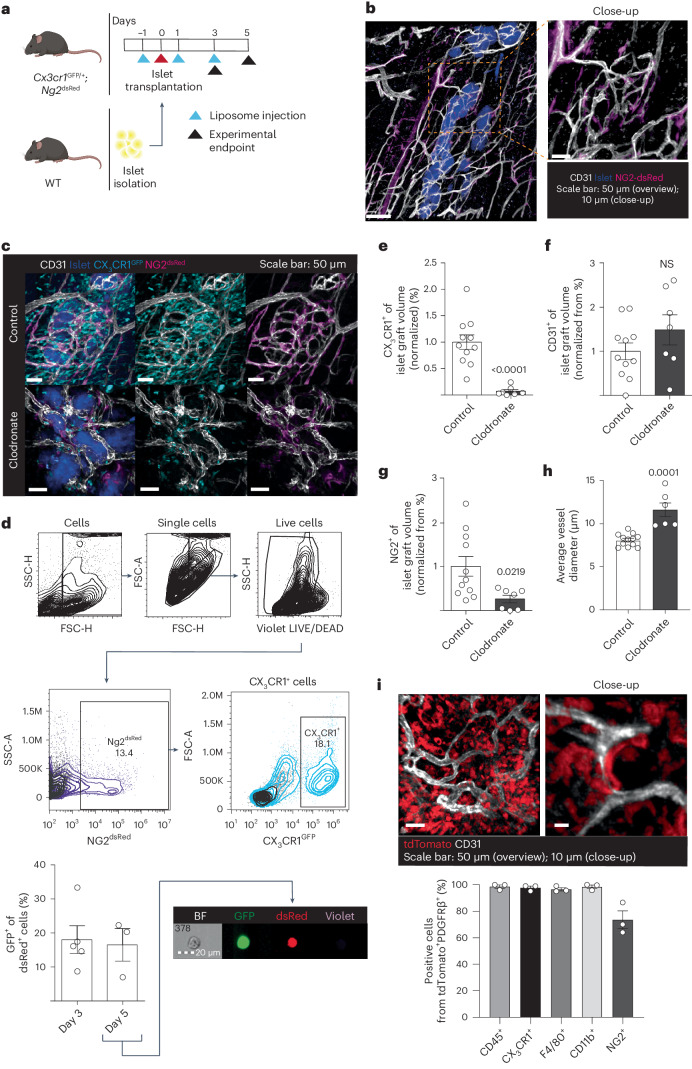
Macrophages align islet vasculature, express mural cell markers and are required for vessel maturation during engraftment. **a**, Experimental layout of the ischemic model of syngeneic islet transplantation. Isolated islets were transplanted into the abdominal muscle of recipient mice (red arrowhead) to enable in vivo imaging of the newly formed vasculature and the cells recruited to the hypoxic sites (black arrowhead). Control or clodronate liposome administration is indicated by the blue arrowhead. **b**, Representative images of engrafted islets (blue) 5 days after transplantation where the newly formed intra-islet CD31^+^ vasculature (white) is lined by NG2^+^ mural cells (magenta) in *Cx3cr1*^*GFP/+*^*;Ng2*^*dsRed*^ recipient mice (three repetitions). **c**, Representative images of islet transplantation sites of control and macrophage-depleted (clodronate) *Cx3cr1*^*GFP/+*^*;Ng2*^*dsRed*^ mice. CD31^+^ vasculature, white; transplanted islets, blue; CX_3_CR1^GFP+^ macrophages, cyan; NG2^dsRed^ mural cells, magenta (11 repetitions for Control, 7 repetitions for Clodronate). **d**, Flow cytometry (gating strategy and quantification) and FlowSight imaging (representative image at day 5) identifies CX_3_CR1-expressing NG2^+^ cells at islet transplantation sites at 3 days and 5 days after transplantation in *Cx3cr1*^*GFP/+*^*;Ng2*^*dsRed*^ mice. Quantification is shown as percentage of CX_3_CR1^GFP+^ cells from total NG2^dsRed^ cells (*n* = 4(Day 3)-3(Day 5)). **e**, Clodronate treatment of *Cx3cr1*^*GFP/+*^*;Ng2*^*dsRed*^ mice resulted in reduced presence of macrophages, as quantified as percentage of CX_3_CR1^+^ volume compared to islet graft volume. **f**, Unchanged vessel areas, as quantified as CD31^+^ volume compared to islet graft volume. Reduced mural cell staining quantified as NG2^+^ volume compared to islet graft volume (**g**) and increased average vessel diameters (**h**) (*n* = 8(Control)-3(Clodronate)). **i**, Representative overview and close-up images of tdTomato^+^ lineage-traced macrophages (red) and CD31^+^ vasculature (white) in engrafted islets 7 d after transplantation into *Cx3cr1*^*CreERT2*^ *Rosa26*-tdTomato lineage-traced mice and characterization of the tdTomato^+^PDGFRβ^+^ intra-islet graft cells by flow cytometry (based on gating strategy in Fig. 1e and Extended Data Fig. 1f) (*n* = 3). Two-tailed unpaired Student’s *t*-test (**f**,**g**). Two-tailed Mann–Whitney *U*-test (**e**,**h**). Data are shown as average ± s.e.m. FSC-A, forward scatter area; FSC-H, forward scattter height; NS, not significant; SSC-H, side scatter height.

**Extended Data Fig. 8:**
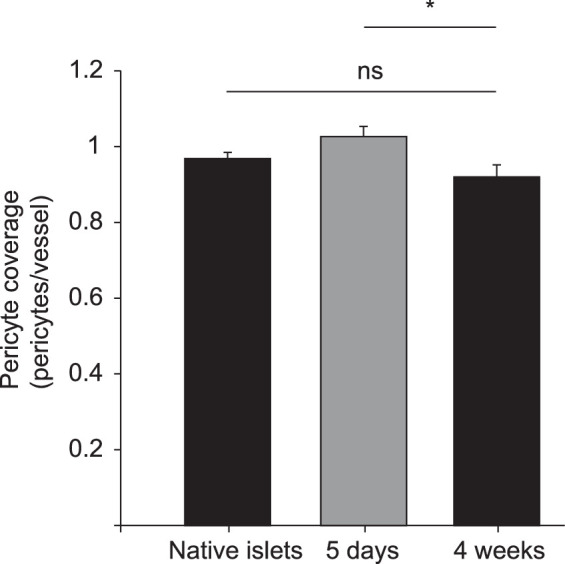
Mural cells are recruited to the newly formed vascultaure of transplanted islet. Mural cells are recruited to the transplanted islet early in the revascularization process. There is no difference between the degree of mural cell coverage (mural cells/vessels) in islets in the healthy pancreas (n = 6) or islets that have engrafted in mouse muscle for 4 weeks (n = 4). At 5 days post-transplantation (n = 6), the number of mural cells over vessels exceed the amount 4 weeks later. One-Way ANOVA followed by Bonferroni’s post-hoc test. Data shown as average ± SEM.

The model of HLI ensures strong hypoxic stimuli and consequent angiogenic response in gastrocnemius muscle, but the anatomical localization of the muscle makes in vivo visualization of newly formed vessels a challenge. To enable in vivo observations of macrophages and mural cells interacting with newly formed vessels, we used our previously developed and characterized model of syngeneic pancreatic islet transplantation to abdominal muscle^2,3,26^. This model results in localized hypoxia within the isolated and transplanted islet grafts, followed by de novo islet revascularization by vessels originating from muscle tissue^2^. By transplanting islets into macrophage reporter and pericyte reporter mice (*Cx3cr1*^GFP/+^;*Ng2*^dsRed^ mice; Fig. 5a), we found that the newly formed intra-islet vessels were fully covered by mural cells by day 5 after transplantation (Fig. 5b and Extended Data Fig. 8). Furthermore, macrophages from the recipient mice were promptly recruited to the transplanted islets (Fig. 5c)^26^. In agreement with our observations in the HLI model, 16.5 ± 4.8% of the recruited macrophages co-expressed the mural cell marker NG2 and the macrophage marker CX_3_CR1 at D5 after islet transplantation (Fig. 5d). Furthermore, using FlowSight (imaging flow cytometry), the NG2 reporter signal was confirmed not to localize to proteasomes (Fig. 5d). To determine if macrophages contribute to mural cell coverage, we administrated clodronate liposomes to deplete macrophages before and after islet transplantation. Clodronate liposome treatment caused a 92.8 ± 0.1% depletion of macrophages in transplanted islets compared to islets transplanted to mice receiving control liposomes (Fig. 5e). Although the treatment did not affect total vascular volumes per islet volume (11.2 ± 2.1% (control), 16.6 ± 3.8% (clodronate)) (Fig. 5f), macrophage depletion resulted in severe deficiency of perivascular NG2^+^ mural cells at the sites of islet transplantation (4.9 ± 1.1% (control), 1.3 ± 0.4% (clodronate)) (Fig. 5g), along with increased vessel diameter indicative of immature vessels (Fig. 5h). To further characterize isolated intra-islet macrophages, islets were syngeneically transplanted to the muscle of mice with lineage-traced macrophages (*Cx3cr1*^*CreERT2*^ × *Rosa26*-tdTomato). As a result, tdTomato-expressing macrophages were found aligning the islet vasculature 5 days after islet transplantation (*n* = 3), and 10.7 ± 4.0% of the tdTomato^+^ cells co-expressed PDGFRβ, as determined by flow cytometry. In addition, 73.5 ± 6.7% of the tdTomato^+^PDGFRβ^+^ macrophages also expressed NG2, but loss of myeloid cell markers could not be detected at this early timepoint (Fig. 5i). These results demonstrate that, in a model of localized hypoxia, as well as in the model of HLI, macrophages express mural cell markers and take on mural cell functions at sites of ischemia.

### Mural cell-like macrophages are required for functional recovery

**Fig. 6:**
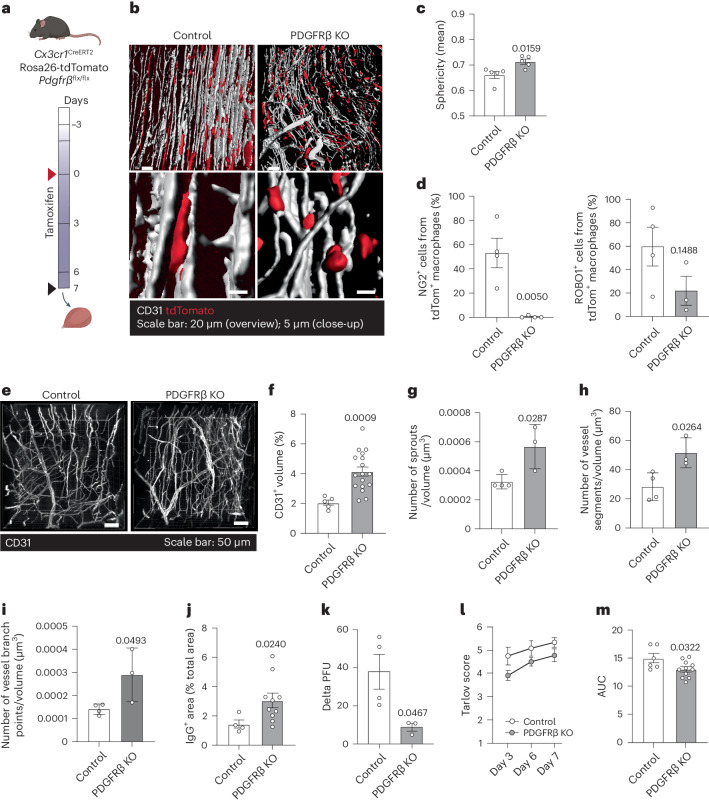
Macrophage deficiency of PDGFRβ prevents their perivascular positioning in ischemic muscles, leads to vessel leakiness and disturbs functional recovery of the ischemic leg. **a**, Experimental layout of tamoxifen-induced PDGFRβ deletion in macrophages in PDGFRβ KO mouse model (*Cx3cr1*^*CreERT2*^ *Rosa26*-tdTomato × *Pdgfrb*^*flx/flx*^). Tamoxifen treatment started 3 days before ischemia and continued throughout the experiment (blue gradient). Red arrowhead marks ischemia induction and black arrowhead in vivo imaging. Control mice are *Rosa26*-tdTomato *Pdgfrb*^*flx/flx*^ and *Pdgfrb*^*flx/flx*^ littermates. **b**, Representative overview and close-up confocal images of CX_3_CR1^+^ macrophages (tdTomato) and CD31^+^ blood vessels in ischemic gastrocnemius muscles demonstrate a marked change in macrophage morphology in mice where PDGFRβ was deleted in their CX_3_CR1-expressing cells (PDGFRβ KO, *Cx3cr1*^*CreERT2*^ *Rosa26*-tdTomato × *Pdgfrb*^*flx/flx*^) compared to the control group. **c**, The sphericity of tdTomato^+^ macrophages was increased in PDGFRβ KO (*Cx3cr1*^*CreERT2*^ *Rosa26*-tdTomato × *Pdgfrb*^*flx/flx*^) mice compared to control group (*n* = 5(Control)-5(PDGFRβ KO)). **d**, The percentage of NG2^+^ cells (left) and ROBO1^+^ cells (right) in the tdTomato^+^ macrophages was reduced in PDGFRβ KO mice (*Cx3cr1*^*CreERT2*^ *Rosa26*-tdTomato × *Pdgfrb*^*flx/flx*^) compared to control mice (*n* = 4). **e**, Representative images of CD31^+^ vasculature (white) in control and PDGFRβ KO (*Cx3cr1*^*CreERT2*^ *Rosa26*-tdTomato × *Pdgfrb*^*flx/flx*^) mice D7 after ischemia (gastrocnemius muscle whole mounts). **f**, The vessel volume quantified as percent of CD31^+^ volume was increased in PDGFRβ KO mice (*Cx3cr1*^*CreERT2*^ *Rosa26*-tdTomato × *Pdgfrb*^*flx/flx*^, *n* = 6) compared to control mice (*n* = 2). The number of sprouts (**g**), vessel segments (**h**) and vessel branch points (**i**) were increased in PDGFRβ KO mice (*Cx3cr1*^*CreERT2*^ *Rosa26*-tdTomato × *Pdgfrb*^*flx/flx*^, *n* = 4) compared to control mice (*n* = 3) 7 d after schemia. Quantification was done using the Filaments plugin in Imaris and normalized by perfused lectin volume. **j**, The vessel leakage (percentage of extravascular IgG^+^ area staining from total image area) was increased in PDGFRβ KO mice (*Cx3cr1*^*CreERT2*^ *Rosa26*-tdTomato × *Pdgfrb*^*flx/flx*^, *n* = 5) compared to control mice (*n* = 5) at D7 after ischemia. The functional recovery of the ischemic leg was reduced in PDGFRβ KO mice (*Cx3cr1*^*CreERT2*^ *Rosa26*-tdTomato × *Pdgfrb*^*flx/flx*^) compared to control mice, as demonstrated by reduced perfusion of the hindlimb assessed by laser speckle flowmetry at day 7 (**k**) and Tarlov score (**l**,**m**). Delta perfusion unit (PFU) represents the difference between PFU at 55 °C and room temperature (**k**, *n* = 4(Control)-3(PDGFRβ KO)). **m**, Area under the curve (AUC) of Tarlov score graph in **l**; *n* = 6(Control)-11(PDGFRβ KO). In **c**–**f** and **j**, each dot represents an area analyzed from tile scan image of muscle whole mounts. In **g**–**i** and **k**–**m**, each dot represents a mouse. Two-tailed unpaired Student’s *t*-test (**d**,**f**–**j**). Two-tailed Mann–Whitney *U*-test (**c**,**k**–**m**). Data are shown as average ± s.e.m.

**Extended Data Fig. 9:**
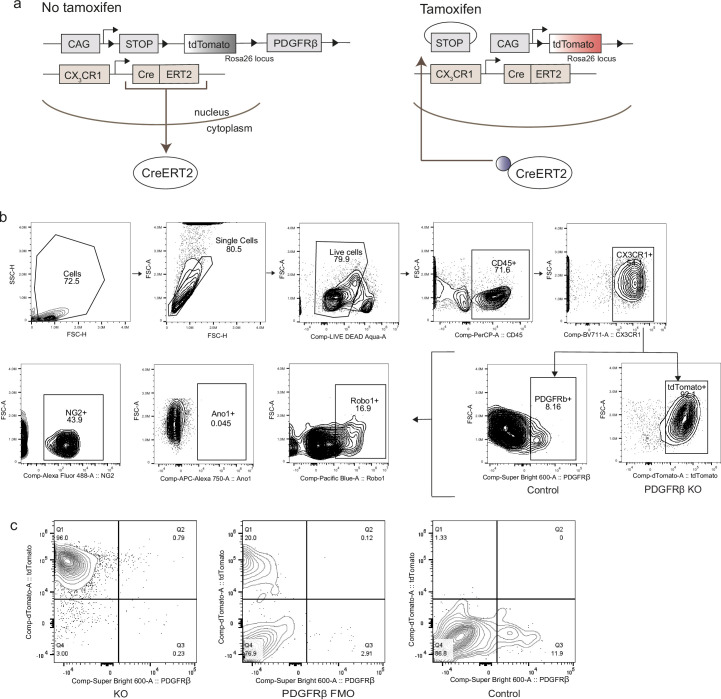
Experimental model of macrophage-specific PDGFRβ depletion. **a**) Schematic representation of the CX_3_CR1^+^ cell specific in PDGFRβ KO mouse model (*Cx3cr1*^CreERT2^ *Rosa26*-tdTomato x *Pdgfrb*^flx/flx^) where heritable labelling by tdTomato and deletion of PDGFRβ in CX_3_CR1-expressing macrophages is induced by tamoxifen treatment. **b**) Gating strategy for macrophages in PDGFRβ macrophage depletion experiments in PDGFRβ KO mouse model (*Cx3cr1*^CreERT2^ *Rosa26*-tdTomato x *Pdgfrb*^flx/flx^, related to Fig. 7d). **c**) Recombinant efficiency of the PDGFRβ KO mouse model (*Cx3cr1*^CreERT2^ *Rosa26*-tdTomato x *Pdgfrb*^flx/flx^) showing PDGFRβ expression in tdTomato^+^ cells. Experimental layout according to the schematic in Fig. 7a.

PDGFRβ-mediated signaling is central to the recruitment of mural cells to blood vessels during angiogenesis and mediates their close contact to the endothelium during homeostasis. To study if PDGFRβ-mediated signaling in macrophages controls their perivascular positioning and mural cell functions in the ischemic muscle, we generated macrophage-specific PDGFRβ knockout mice (*Cx3cr1*^CreERT2^ *Rosa26*-tdTomato × *Pdgfrb*^flx/flx^ mice, hereafter referred to as PDGFRβ KO mice). As a result, tamoxifen administration leads to a deletion of PDGFRβ specifically in CX_3_CR1^+^ macrophages (Fig. 6a and Extended Data Fig. 9a). To ensure high tamoxifen-induced recombination, both the daily dose and time of treatment were doubled compared to the lineage tracing experiments. Thus, peroral administration of tamoxifen (2 mg) twice a day for 10 days resulted in high recombination efficacy, as 85.0 ± 5.5% of all CX_3_CR1^+^ cells expressed tdTomato, and also did not express PDGFRβ (Extended Data Fig. 9b,c). Macrophage-specific PDGFRβ KO resulted in loss of the elongated macrophage shape in the ischemic muscle (Fig. 6b,c), demonstrating that PDGFRβ-mediated signaling contributes to the mural cell morphology of macrophages. Furthermore, flow cytometry analysis of PDGFRβ KO macrophages demonstrated that NG2 was not at all upregulated, and levels of ROBO1 showed a tendency toward reduction (Fig. 6d and Extended Data Fig. 9b). This demonstrates that macrophage expression of PDGFRβ is required for them to take on expression of other mural cell-associated markers. The capillary bed within ischemic gastrocnemius muscles was then investigated in the *Cx3cr1*^CreERT2^ × *Rosa26*-tdTomato × *Pdgfrb*^flx/flx^ mice using immunohistochemistry of whole mounts (Fig. 6e) to assess angiogenesis as well as vessel functionality. In the PDGFRβ KO mice D7 after ischemia, vessel densities, number of sprouts, vessel segments and branch points as well as vessel permeability were all increased in the gastrocnemius muscle of PDGFRβ KO mice D7 after ischemia (Fig. 6f–j), demonstrating that PDGFRβ deletion in macrophages results in formation of immature vasculature in the ischemic muscle. Consequently, blood perfusion of footpads of the affected leg was demonstrated to be further impaired in PDGFRβ KO mice D7 after HLI induction (Fig. 6k). Furthermore, assessment of the functional recovery of the limb using Tarlov scoring revealed reduced recovery of function in PDGFRβ KO mice (Fig. 6l,m), demonstrating an important role of macrophage PDGFRβ in the healing of ischemic injuries. Together, these results demonstrate that PDGFRβ expression in macrophages in ischemic muscles is essential for initiating a phenotype switch toward mural cells, which is crucial for blood flow recovery after injury.

## Discussion

Here we report that macrophages in adult ischemic tissue undergo a cellular program to morphologically, transcriptomically and functionally resemble mural cells while downgrading their macrophage identity. This phenotype switch was dependent on induced macrophage expression of PDGFRβ, and impaired macrophage-specific PDGFRβ signaling strongly compromised the recruitment of macrophages to vasculature, which consequently compromised blood vessel maturation and function at the affected site. Thus, macrophage phenotype conversion was crucial for the re-establishment of functional tissue perfusion necessary for healing and prompts exploration when developing immunotherapies to restore tissue function after ischemic insults.

Macrophages are dynamic tissue-resident immune cells with a wide arsenal of important functions in both host defense and tissue restoration after injury^27^. The local microenvironment greatly influences macrophage effector functions, resulting in the same cell exerting different tasks at different timepoints^27^. Thus, macrophages entering an infected or injured area adopt an inflammatory phenotype after activation by interferon gamma and/or PAMPs and DAMPs (pathogen-associated or danger-associated molecular patterns, respectively), facilitating clearance of bacteria and cell debris. When this acute phase is over, the inflammatory phenotype of macrophages is dampened as they shift into a restorative phenotype driven by specific cytokines (IL-4/IL-13)^28^, tissue-specific defense collagens^29^ or efferocytosis of apoptotic neutrophils^30^. In addition, the influence of metabolic factors on macrophage activation is increasingly recognized^4^. Stabilization of HIF-1α during hypoxia coordinates transcription of pro-inflammatory and glycolytic pathways in macrophages^31^. In contrast, IL-4/IL-13-activated macrophages undergo a metabolic switch by upregulating genes important for fatty acid oxidation, which occurs in parallel with a functional shift from pro-inflammatory macrophages into tissue restorative at the injured site^32^. In injured muscle from patients with myocardial infarction or peripheral arterial disease, as well as in mouse models of HLI, macrophages have been found to chaperone the vasculature and attain a mural cell morphology^11^. In the present study, we found that these perivascular macrophages expressed proteins associated with mural cell identity while reducing their expression of those associated with macrophages. Intriguingly, two studies of embryonic development identified mural cell marker expression of macrophages in skin and brain using immunohistochemistry^33,34^. Thus, Yamazaki et al.^34^ showed that F4/80^+^ embryonic myeloid progenitors contribute to the mural cell pool of the skin in mouse embryos. In addition, a capacity of CD31^+^F4/80^+^ macrophages in the central nervous system to differentiate toward NG2/PDGFRβ/desmin-expressing cerebrovascular mural cells early during development was demonstrated^33^. Importantly, these observations of embryonic tissues were based merely on immunohistochemistry and not further characterized at transcriptomic level, which precludes conclusions of potential transdifferentiation.

Cellular transdifferentiation can be studied by genetic fate mapping followed by single-cell sequencing of lineage-traced cells. Using this approach, we identified a subpopulation of lineage-traced macrophages that had initiated expression of mural cell markers while downregulating their innate immunity signature at 3 weeks after ischemia induction. The collective term ‘mural cells’ refers to pericytes and smooth muscle cells as they cannot be separated by specific and stable marker genes and have functional overlap in regard to vessel maturation and function^12,35^. A closer investigation of the gene expression profiles revealed that the subpopulation of macrophages in which various mural cell genes were progressively expressed to higher degree (for example, *Pdgfrb*, *Sema5a* and *Adamts2*) in parallel presented lower expression of characteristic macrophage markers (for example, *Ptprc*, *Cx3cr1* and *Mrc1*). Interestingly, iNOS expression was not significantly induced in the mural cell-like macrophages, indicating that the recent observation of macrophages adopting blood flow regulation in the ischemic muscle refers to another set of macrophages^11^. Furthermore, the transcriptomic shift of macrophages toward mural cells was further enhanced when unspliced transcripts were included, which was selectively high in proportion in the macrophage population expressing mural cell genes. Unspliced reads can reflect an ongoing induction of gene expression and future cell state^17^ and could, therefore, indicate that this macrophage population has not reached its final state and is further transitioning toward the mural cell phenotype. Mural cell ontogeny is known to be heterogeneous and varies depending on tissue and context^36^. The predominant view is that local proliferation of pericytes is induced after injury, as shown by upregulation of genes involved in mitotic cell division^23^. Likewise, neointimal smooth muscle cells have been shown to undergo phenotypic switching and consequent cell proliferation in response to vascular injury^37,38^. This study demonstrates that mural cell proliferation in the ischemic muscle is accompanied by a macrophage-toward-mural cell switch. This represents a previously undescribed macrophage capability, as phenotype switch and consequent transdifferentiation of macrophages into mural cells, or any other cell type, has not been reported previously in adult tissue. Considering the inherent ability of immune cells to migrate to and quickly accumulate at sites of injury, the important role of macrophages in assuming mural cell functions to promote tissue injury aligns with their characteristic behavior in response to physiological insults.

Mural cells promote vessel maturation as well as provide structural support and integrity to the vessel wall, which are crucial for vascular function. In the developmental setting, the PDGF-BB:PDGFRβ axis is central for mural cell recruitment to angiogenic vessels, and knockout mouse models for either gene present with severe mural cell deficiency and concomitant vascular dysfunction with leaky and dilated vessels that results in embryonic or perinatal lethality^13,15^. Here we show that the same signaling pathway is important to retain PDGFRβ^+^ macrophages at a perivascular location in the ischemic muscle and that depletion of macrophages during post-ischemic angiogenesis results in formation of dilated vessels with reduced mural cell coverage. Furthermore, when PDGFRβ was depleted in macrophages in the HLI model, vessel maturation was reduced, resulting in increased vascular leakiness, reduced perfusion and impaired functional recovery. This demonstrates that macrophages take on the function of mural cells after ischemic injury. As others and we have found that macrophage recruitment to angiogenic vessels occurs in a CXCL12-dependent manner^11,39^, we now propose that macrophage expression of PDGFRβ and concomitant PDGFRβ signaling is necessary for proper adhesion and organization of macrophages along the vessels, as reported for pericytes^40^. In addition, we demonstrated that macrophage-specific expression of PDGFRβ is an important regulator of vascular density and integrity in the site of ischemic injury. During tissue restoration after ischemic injury, the macrophages thereby both shift toward a mural cell profile as well as undertake several mural cell functions of importance for healing of ischemic injuries.

Peripheral vascular disease and ischemic heart disease are the most common cardiovascular disorders and develop as a result of loss of tissue function due to inadequate restoration of tissue perfusion after the ischemic events^41^. Efficient treatment strategies are still lacking, and several different approaches are currently being assessed. Local stimulation of angiogenesis to re-establish perfusion of the injured tissue has been thoroughly investigated by local upregulation of growth factors and chemokines, including VEGF, HGF and SDF-1, alone or co-delivered with, for example, PDGF-BB, but has so far showed limited clinical success as the new blood vessels are leaky and immature^42–44^. More recently, immune-based therapeutic approaches encompassing biomaterial, biologics and targeted cell and gene therapy have been explored to limit injury and stimulate regain of tissue function by modifying specific immune responses important for the endogenous repair program. Of these, in vivo-generated anti-fibrotic chimeric antigen receptor (CAR) T cells have demonstrated promising results in mouse models of cardiac injury^45,46^. Interestingly, pharmacological induction of pericyte differentiation into vascular smooth muscle cells was shown to improve arteriologenesis and functional recovery of the infarcted murine heart^47^. Whether the current findings of an endogenous macrophage-to-mural cell switch being important for the healing of ischemic injury can be therapeutically enhanced requires further explorations.

Together, our data reveal that a subpopulation of lineage-traced macrophages in the ischemic muscle initiates a gene and protein expression profile extensively overlapping with the functional characteristics of mural cells while downgrading a macrophage gene signature. This cellular switch was shown to be central for vascular maturation and function and pinpoints macrophages as a potential target for therapeutically enhancing vascular integrity and healing of ischemic injuries.

## Methods

### Experimental animals and lineage tracing experiments

All animal experiments were approved by the Uppsala Region Laboratory Animal Ethics Board (numbers C81/14 and C12740/20).

C57Bl/6J mice (C57BL/6JBomTac, Taconic Biosciences), *Cx3cr1*^CreERT2^ mice (B6.129P2©-Cx3cr1^tm2.1(cre/ERT2)Jung^/J, RRID: IMSR_JAX:020940 (refs. ^48,49^)), *Rosa26*^tdTomato^ mice (Ai14, B6.Cg-Gt(ROSA)26Sor^tm14(CAG-tdTomato)Hze^/J, RRID: IMSR_JAX:007914 (ref. ^49^)), *Pdgfrβ*^eGFP^ mice (Tg(Pdgfrb-EGFP)JN169Gsat/Mmucd, RRID: MMRRC 031796-UCD)^50^, *Cx3cr1*^GFP/+^ mice (B6.129P2(Cg)-Cx3cr1^tm1Litt^/J, RRID: IMSR_JAX:005582 (ref. ^51^)), *Pdgfrβ*^*flx/flx*^ mice, kindly provided by Christer Betsholtz (Uppsala University) and *Ng2*^dsRed^ mice (Tg(Cspg4-DsRed.T1)1Akik/J, RRID:IMSR_JAX:008241 (ref. ^52^)) were used in the present study. All animals used were males and females mixed, 7–12 weeks old. Mice were kept as a rule in groups of five per cage and two per cage after major surgery. The plastic cages had a layer of wood shavings covering the floor and contained bedding enrichment. Cages, food and water bottles were changed twice a week, and supervision was carried out daily. CreER^T2^-mediated recombination was initiated by oral gavage of 2 mg of tamoxifen (Sigma-Aldrich) in 90% of corn oil and 10% of ethanol given once every 24 h for 5 days for lineage tracing and every 12 h for 10 days to the *Pdgfrβ*^*flx/flx*^ mice.

### HLI

Mice were anesthetized (isoflurane, 2.5%, Forane, Abbott Laboratories), and the fur was removed from the left leg. HLI was induced as described previously^53^. In brief, the left femoral artery was separated from the femoral vein and nerve, followed by ligation and cutting of the femoral artery above the superficial epigastric artery branch. Postoperative pain relief was achieved with subcutaneous administration of carprofen (5 mg kg^−1^, Rimadyl Bovis vet., 014920, Zoetis). Laser speckle flowmetry was carried out on day 1 to confirm HLI. Functional recovery of limb function was scored over time based on the following Tarlov scoring system: 0: no movement; 1: barely perceptible movement, no weight bearing; 2: frequent and vigorous movement, no weight bearing; 3: supports weight, may take one or two steps; 4: walks with only mild deficit; 5: normal but slow walking; and 6: full and fast walking^54^. Healthy controls are defined as animals who have not undergone HLI induction.

### Laser speckle flowmetry

Blood flow in footpads was measured as previously described in ref. ^11^. In brief, anesthetized (spontaneous inhalation of 2.5% isoflurane, Forane, Abbott Laboratories) mice were placed in a prone position with paws resting on tubing with circulating water (laser speckle contrast analysis, 785-nm laser, 20-µm resolution, PeriCam HR PSI System, Perimed). After baseline registration, the circulating water temperature was increased, resulting in an approximately 10 °C temperature increase on the dorsal side of the footpad. Blood flow was recorded for 2 min after reaching the plateau, at 10 images per second, and analyzed in PIMSoft (Perimed).

### Pancreatic islet isolation and transplantation

Islets were isolated from donor C57Bl/6J mice using the following protocol^55^. Mice were euthanized by cervical dislocation, and, thereafter, an ice-cold Collagenase A solution from *Clostridium histolyticum* (2.5 mg ml^−1^, 10103578001, Roche Diagnostics) in HBSS (24020-091, Gibco) was injected into the pancreas via the common bile duct. The pancreas was then removed and placed in a 37 °C water bath for 18 min. After tissue digestion, islets were separated from the exocrine fraction by density gradient centrifugation (Histopaque-1077, 10771, Sigma-Aldrich, and RPMI 1640, 21875-034, Gibco) and hand-picking of intact islets as evaluated by edge clarity. The free-floating islets were incubated overnight at 37 °C in islet culture medium (RPMI 1640 medium, GlutaMAX Supplement, 61870036, Gibco) with added D-glucose (11.1 mM; G7528, Sigma-Aldrich), FBS (10%, 16000044, Gibco) and penicillin–streptomycin solution (L0022, BIO-WEST).

Immediately before transplantation, islets were fluorescently labeled with the intracellular probe CellTracker Blue CMAC (C2110, Invitrogen) according to the manufacturer’s instructions. Labeled islets were transplanted through a butterfly needle to the abdominal external oblique muscle of syngeneic *Cx3cr1*^GFP/+^ *Ng2*^dsRed^ mice anesthetized with isoflurane (2.5%, Forane, Abbott Laboratories)^2^.

### Macrophage depletion strategy

Macrophage depletion was achieved by administration of clodronate liposomes (Liposoma Research) in the tail vein (500 µg) and/or intramuscularly (125 µg). Control liposomes were given to the control group following the same protocol. Efficiency of depletion was determined by in vivo detection (intravital microscopy) of CX_3_CR1^+^ cell density at the site of islet engraftment and by flow cytometry–based quantification of macrophages (CD45^+^CD11b^+^CX_3_CR1^+^Ly6C^−^) in the gastrocnemius muscle.

### Single-cell isolation

The mice were euthanized by cervical dislocation, after which the gastrocnemius muscle or the islet graft was removed and minced into very small pieces with a scalpel. The tissue was then mechanically and enzymatically dissociated as follows. The muscle was incubated in Collagenase II (500 U ml^−1^) (17101015, Gibco) in RPMI 1640 (21875-034, Gibco) in 37 °C for 30 min during intermittent pipetting. After washing the tissue with ice-cold DPBS (14190-094, Gibco), it was incubated for a further 30 min with Collagenase IV (15 U ml^−1^) (17104019, Gibco) and dispase (2.4 U ml^−1^) (17105041, Gibco) in RPMI 1640, with intermittent pipetting. Thereafter, the tissue suspension was passed through a 23-gauge 0.6-mm needle 8–10 times and filtered through a 70-µm cell strainer.

### Flow cytometry

For flow cytometry, single-cell suspensions were subjected to debris removal (130-109-398, Miltenyi Biotec), followed by incubation with 10% FBS (16000044, Gibco) in RPMI 1640 (21875-034, Gibco) for 20 min on ice. Next, extracellular antigen fluorochrome-conjugated primary antibodies were added for 15 min on ice, with appropriate control antibodies (Extended Data Table 1). Finally, live/dead cell staining was carried out using CellTrace Calcein Violet, AM (C34858, Invitrogen) or LIVE/DEAD Fixable Violet Dead Cell Stain Kit (L34964, Invitrogen), according to the manufacturer’s instructions. Cells were analyzed using a CytoFlex S (Beckman Coulter) flow cytometer with CytExpert software (Beckman Coulter) or Cytek Northern Lights in the three-laser configuration (16V-14B-8R), and data analysis was performed using FlowJo software (BD Biosciences).

For FlowSight, single-cell suspension was stained with CellTrace Calcein Violet, AM (C34858, Invitrogen) according to the manufacturer’s instructions. A FlowSight imaging flow cytometer (EMD Millipore) was used to acquire images of the cells. Cells were gated from debris using a plot of bright-field channel area versus bright-field channel aspect ratio. Image analysis was performed in IDEAS software (EMD Millipore).

A PrimeFlow RNA assay was used to measure *Pdgfrβ* mRNA by flow cytometry in parallel with immunolabeling and was performed as previously described^56^. PDGFRβ probe (VB6-19706-PF, Thermo Fisher Scientific) was used for testing the presence of *dgfrβ* mRNA; ACTB probe (VB1-10350-PF, Thermo Fisher Scientific) was used to attest the efficiency of the procedure; and dapB (VF4-10408-PF, Thermo Fisher Scientific) was used as negative control. Single-cell suspensions of muscles were stained with surface markers for myeloid markers as well as PDGFRβ. Intracellular staining for GFP was performed with anti-GFP conjugated to FITC to combat potential loss of fluorescence during the performance of the PrimeFlow protocol.

### scRNA-seq, data processing and analysis

A total of 755 tdTomato^+^PDGFRβ^+/−^ (tdTomato^+^PDGFRβ^+^ and tdTomato^+^PDGFRβ^−^) cells and 372 tdTomato^−^PDGFRβ^+^ cells from ischemic muscles at day 21 after ischemia onset, and a total 190 tdTomato^+^PDGFRβ^+/−^ cells and 192 tdTomato^−^PDGFRβ^+^ cells from healthy muscle, were FACS sorted into Smart-seq2 plates in two batches using a BD FACSAria III cell sorter in a four-laser configuration (405 nm, 488 nm, 561 nm and 633 nm) (BD Biosciences). RNA libraries were prepared using the Smart-seq2 protocol^57^, and the two batches were sequenced with an Illumina HiSeq 2000 at the Eukaryotic Single Cell Genomics Facility at SciLifeLab, Stockholm, at two distinct timepoints. Data from healthy muscle and muscle at day 21 after ischemia were used for processing of the raw sequencing data, whereas only day 21 processed data were used for analysis of the clusters.

The raw sequencing data were converted to demultiplexed FASTQ files using deindexer (https://github.com/ws6/deindexer) based on the Nextera index adapters and the 384-well layout. An average depth of 0.6 million reads (43 bp, single end) per cell was obtained. Reads were then aligned to the mouse genome (GRCm38) using STAR version 2.7.7a (ref. ^58^), and featureCounts version 2.0.1 (ref. ^59^) was subsequently used to extract raw gene expression count matrix from the alignments.

Scanpy version 1.7.1 package^60^ was used to convert the cell by gene count matrix to AnnData object and for further analysis of the scRNA-seq data. Cell libraries were filtered out if (1) the percentages of mitochondrial and ribosomal gene families were above 10%; (2) the number of unique genes in a cell was less than 500; and (3) predicted doublet by Scrublet version 0.2.3 (ref. ^61^). In total, 1,425 cells were retained for downstream analysis. Genes detected in fewer than three cells along with mitochondrial genes were excluded from the analysis, leaving 38,178 genes in the count matrix.

The Python version of Velocyto version 0.17.17 (ref. ^17^) run_smartseq2 command was used to count unspliced transcripts from the alignments masking out expressed repeat annotation downloaded from UCSC genome browser. The resulting loom file was read, and the unspliced counts were added to AnnData object in ‘layers’ using scvelo version 0.2.4 (ref. ^62^).

Raw counts were then normalized and log transformed and were further processed for identification of highly variable genes (HVGs) using flavor ‘cell_range’^63^, and only the HVGs were used for downstream dimensionality and visualization computation. Data were then scaled to be zero centered and linear data compression using principal component analysis (PCA) from which the top 40 principal components (PCs) explaining about 70% of the variance in the dataset were chosen for downstream analysis. Graph construction was also run on the top 40 PCs using approximate *k*-nearest neighbor (KNN)^64^ search with *k* = 15. Nonlinear dimensionality reduction was run on the top 40 PCs using UMAP^65^ and embedded into two final dimensions. Because the single-cell data were obtained and sequenced at two different timepoints, we used Scanorama version 1.7 (ref. ^66^) to integrate the two datasets to correct for the introduced batch effect.

Unsupervised graph clustering was run using the Leiden method^67^ with modularity resolution parameter (res = 0.4). Macrophage cluster was further split into clusters 0, 1 and 2 using the Leiden method with res = 0.3. Cell type annotation from ref. ^68^ was used as reference, and R Seurat version 3 ‘FindTransferAnchors’ and ‘TransferData’ methods were applied for cell type prediction^69^. The marker genes used in the reference were further investigated to validate the predictions. For boundary cases where the cluster and prediction did not agree, cell type annotations were changed to follow the majority of cells in the cluster.

Differential gene expression analysis between clusters was performed using Scanpy ‘rank_genes_groups’ with Wilcoxon rank-sum test based on normalized and logarithmized raw gene expression considering all clusters together. Retrieved DEGs (*P*_adj_ ≤ 0.05) were divided into upregulated and downregulated genes, and the corresponding gene names were loaded to STRING version 11.0b (ref. ^22^) for gene set enrichment analysis. Trajectory inference analysis was performed using PAGA^70^. Pseudotemporal orderings were constructed by selecting cluster 0 cells as root. Diffusion pseudotime (DPT) was calculated for all the remaining cells relative to the root. Cellular trajectories were assembled for paths through specified clusters, with cells ordered by DPT values.

Gene unspliced ratio was calculated by dividing the unspliced counts by the sum of the unspliced counts and the spliced counts from featureCounts, which is then aggregated over cells within a given cluster, and the mean value was taken. Histograms of the mean unspliced ratios revealed a cutoff at 0.3, when taking genes with ≥0.3 mean unspliced ratio omitting lowly expressed genes in the majority of the cells in the cluster. The total number of the unspliced counts of a given cluster divided by the total sum of the unspliced and spliced counts was then calculated for the remaining genes. This score was used as the final gene unspliced ratio, and a cutoff at ≥0.8 was applied to define genes with high unspliced ratio per cluster. The list of genes with high unspliced ratio from cluster 2 was loaded to STRING version 11.0b (ref. ^22^) for gene set enrichment analysis.

### In vivo imaging

Anti-CD31 antibody tagged with Alexa Fluor 647 (102416, BioLegend) was intravenously administered to the mice via the tail vein. Thereafter, mice were anesthetized (isoflurane, 2%, Forane, Abbott Laboratories), and the left gastrocnemius muscle or abdominal external oblique muscle was exposed and mounted using the in-house developed light vacuum window system^71^ for intravital microscopic imaging (Leica SP8).

### Immunohistochemistry

For quantifying vessel perfusion, 50 μg of lectin SBA from Glycine Max (L32462, Invitrogen) was injected intravenously. After 15 min, the mouse was euthanized using cervical dislocation. The whole gastrocnemius muscle was removed and snap frozen using liquid nitrogen. Thereafter, 20-µm-thick cryosections were prepared and fixed for 10 min in ice-cold methanol and incubated for 60 min with 10% FBS (16000044, Gibco) in DPBS (14190-094, Gibco). After this, sections were stained with anti-CD31 antibody (Extended Data Table 1). For detection of tdTomato and GFP proteins, whole gastrocnemius muscles were processed as described previously^72^. In brief, dissected muscles were fixed and permeabilized for 2 h at room temperature in 2% paraformaldehyde (28908, Thermo Fisher Scientific) with 0.1% Triton X-100 (T8787, Sigma-Aldrich), followed by incubation in 15% sucrose overnight at 4 °C. The next day, the muscles were snap frozen using liquid nitrogen/2-methylbutane double-bath as described previously^73^. Thereafter, 20-µm-thick cryosections were prepared and incubated for 60 min with 10% FBS (16000044, Gibco) in DPBS (14190-094, Gibco) and stained (Extended Data Table 1). Vessel permeability was assessed in 0.5% Triton X-100 (T8787, Sigma-Aldrich) permeabilized 20-µm-thick cryosections of gastrocnemius muscles as extravascular IgG (AF568 donkey-anti-mouse IgG; Thermo Fisher Scientific, A10037). Confocal imaging was performed with an LSM700 (Zeiss) and an SP8 (Leica). Image analysis was performed using Imaris software (Oxford Instruments).

### Inclusion and ethics statement

Data are reported in conformity with Animal Research: Reporting of In Vivo Experiments (ARRIVE) guidelines.

### Statistical analysis

Data are presented as mean ± s.e.m., and statistical analysis was performed using GraphPad Prism 9 (GraphPad Software). Outliers were identified using Grubb’s test (alpha = 0.05) and excluded. The Shapiro–Wilk test was used to assess normal distribution. *F*-test and Brown–Forsythe tests were used to determine equal variance when analyzing two or more groups, respectively. In case of fewer than six data points per group, a non-parametric test was used. Unpaired two-tailed Student’s *t*-test was used for comparing two normally distributed groups with equal variance. One-way ANOVA with Dunnett’s post hoc test was used for comparing data with more than two normally distributed groups. The Mann–Whitney *U*-test was used for comparing two groups with either non-normal distribution or non-equal variance. When comparing more than two groups, Kruskal–Wallis with Dunn’s multiple-comparison post hoc test was used. *P* values ≤ 0.05 were considered statistically significant.

### Reporting summary

Further information on research design is available in the Nature Portfolio Reporting Summary linked to this article.

### Supplementary information

Reporting Summary

Supplementary Table 1.

Supplementary Table 2.

Supplementary Table 3.

## Data Availability

Single-cell RNA sequencing raw and processed data are deposited in the Gene Expression Omnibus under accession number GSE211550. All other data that support the findings of this study are available at https://figshare.com/s/fac0a9b0287499cb52c2.

## Extended data

**Extended Data Table 1:** Flow cytometry and immunofluorescence staining antibodies

Antigen	Primary antibody	Concentration	Isotype control	Secondary antibody (if applicable)	Experiment
**CD45**	103137 (Clone 30-F11, BioLegend)	1 ng µl^−1^	400646 (Clone RTK4530, BioLegend)	NA	Flow cytometry
**CX**_**3**_ **CR1**	149033 (Clone SA011F11, BioLegend)	1 ng µl^−1^	400266 (Clone MOPC-173, BioLegend)	NA	Flow cytometry
**CX**_**3**_ **CR1**	149031 (Clone SA011F11, BioLegend)	1 ng µl^−1^	NA	NA	Flow cytometry
**CX**_**3**_ **CR1**	149029 (Clone SA011F11, BioLegend)	1 ng µl^−1^	NA	NA	Flow cytometry
**CD11b**	562287 (Clone M1/70, BD)	1 ng µl^−1^	562308 (Clone A95-1, BD)	NA	Flow cytometry
**CD11b**	101245 (Clone M1/70, BioLegend)	1 ng µl^−1^	NA	NA	Flow cytometry
**CD11b**	563015 (Clone M1/70, BD)	1 ng µl^−1^	NA	NA	Flow cytometry
**Ly6C**	560596 (Clone AL-21, BD)	0.4 ng µl^−1^	560571 (Clone R4-22, BD)	NA	Flow cytometry
**Ly6C**	755204 (Clone HK1.4, BD)	1 ng µl−1	NA	NA	Flow cytometry
**Collagen IV**	ab6586 (Abcam)	10 µg ml^−1^	ab172730 (Clone EPR25A, Abcam)	A31573 (Invitrogen)	IHF
**CD31**	16-0311-85 (Clone 390, Invitrogen)	10 µg ml^−1^	ab18450 (Clone RTK2758, Abcam)	A21247 (Invitrogen)	IHF
**GFP**	600-402-215 (Rockland)	10 µg ml^−1^	NA	NA	IHF
**PDGFRβ**	14-1402-82 (Clone APB5, Invitrogen)	5 µg ml^−1^	NA	712-165-150 (Jackson ImmunoResearch)	IHF
**PDGFRβ**	130-105-323 (Clone REA363, Miltenyi Biotec)	7.5 µg ml^−1^	NA	NA	Flow cytometry
**PDGFRβ**	63-1402-82 (Clone APB5, eBioscience)	1 ng µl^−1^	NA	NA	Flow cytometry
**F4/80**	123137 (Clone BM8, BioLegend)	1 ng µl^−1^	NA	NA	Flow cytometry
**F4/80**	743282 (Clone T45-2342, BD)	1 ng µl^−1^	NA	NA	Flow cytometry
**MERTK**	747893 (Clone 108928, BD)	1 ng µl^−1^	NA	NA	Flow cytometry
**CD64**	741024 (Clone X54-5/7.1, BD)	1 ng µl^−1^	NA	NA	Flow cytometry
**CD49d**	740661 (Clone R1-2, BD)	1 ng µl^−1^	NA	NA	Flow cytometry
**LYVE-1**	14-0443-82 (Clone ALY7, Invitrogen)	1 ng µl^−1^	NA	Conjugated with A20189 (Invitrogen)	Flow cytometry
**CD169**	566604 (Clone 3D6, BD)	1 ng µl^−1^	NA	NA	Flow cytometry
**Timd4**	752025 (Clone RMT4-54, BD)	1 ng µl^−1^	NA	NA	Flow cytometry
**Robo1**	ab274385 (Clone EPR23699-159, Abcam)	1 ng µl^−1^	NA	Conjugated with P30013 (Invitrogen)	Flow cytometry
**Ano1**	ab64085 (Clone SP31, Abcam)	1 ng µl^−1^	NA	Conjugated with A20189 (Invitrogen)	Flow cytometry
**NG2**	ab306569 (Clone EPR23752-147, Abcam)	1 ng µl^−1^	NA	NA	Flow cytometry
**NG2**	ab307168 (Clone EPR23752-147, Abcam)	1 ng µl^−1^	NA	NA	Flow cytometry

## Code availability

Single-cell RNA sequencing data processing and analysis were compiled into a Python notebook and are available at a personal GitHub deposit (https://github.com/feifei/macro_smartseq2).
