# Supplementary material for: Macrophages upregulate mural cell-like markers and support healing of ischemic injury by adopting functions important for vascular support
Source: Nat Cardiovasc Res. 2024 Jun 6;3(6):685–700. doi: 10.1038/s44161-024-00478-0 (PMC11358018; doi:10.1038/s44161-024-00478-0)
Supplement: Supplementary file 1 — Reporting Summary [file 44161_2024_478_MOESM1_ESM.pdf]

nature portfolio

Corresponding author(s): Mia Phillipson

Last updated by author(s): Feb 19, 2024

Reporting Summary

Nature Portfolio wishes to improve the reproducibility of the work that we publish. This form provides structure for consistency and transparency in reporting. For further information on Nature Portfolio policies, see our Editorial Policies and the Editorial Policy Checklist.

Statistics

For all statistical analyses, confirm that the following items are present in the figure legend, table legend, main text, or Methods section.

n/a	Confirmed	
☐	☒	The exact sample size (*n*) for each experimental group/condition, given as a discrete number and unit of measurement
☐	☒	A statement on whether measurements were taken from distinct samples or whether the same sample was measured repeatedly
☐	☒	The statistical test(s) used AND whether they are one- or two-sided<br>*Only common tests should be described solely by name; describe more complex techniques in the Methods section.*
☐	☒	A description of all covariates tested
☐	☒	A description of any assumptions or corrections, such as tests of normality and adjustment for multiple comparisons
☐	☒	A full description of the statistical parameters including central tendency (e.g. means) or other basic estimates (e.g. regression coefficient) AND variation (e.g. standard deviation) or associated estimates of uncertainty (e.g. confidence intervals)
☒	☐	For null hypothesis testing, the test statistic (e.g. *F*, *t*, *r*) with confidence intervals, effect sizes, degrees of freedom and *P* value noted<br>*Give P values as exact values whenever suitable.*
☒	☐	For Bayesian analysis, information on the choice of priors and Markov chain Monte Carlo settings
☒	☐	For hierarchical and complex designs, identification of the appropriate level for tests and full reporting of outcomes
☒	☐	Estimates of effect sizes (e.g. Cohen's *d*, Pearson's *r*), indicating how they were calculated

Our web collection on statistics for biologists contains articles on many of the points above.

Software and code

Policy information about availability of computer code

Data collection	PeriCam HR PSI System with PIMSoft software (Perimed)<br>CytoFlex S flow cytometer with CytExpert software (Beckman Coulter)<br>Cytek® Northern Lights™ with SpectroFlo® software (Cytek)<br>FlowSight imaging flow cytometer (EMD Millipore)<br>Leica SP8 with LasX software (Leica Microsystems)<br>LSM700 with ZEN microscopy software (Zeiss)<br>BD FACSAria III Cell Sorter with BD FACSDiva software (BD Bioscences)
Data analysis	FlowJo software (BD) for flow cytometry<br>Imaris software (Oxford Instruments) for in vivo imaging and immunohistochemistry<br>Jupyter Notebook (Project Jupyter) for Single-cell RNA-seq data processing and analysis<br>Microsoft Excel (Microsoft Office, Microsoft Coorporation) and GraphPad Prism (GraphPad Software) for statistical analysis and graph production.

For manuscripts utilizing custom algorithms or software that are central to the research but not yet described in published literature, software must be made available to editors and reviewers. We strongly encourage code deposition in a community repository (e.g. GitHub). See the Nature Portfolio guidelines for submitting code & software for further information.

Data

Policy information about availability of data

All manuscripts must include a data availability statement. This statement should provide the following information, where applicable:

- Accession codes, unique identifiers, or web links for publicly available datasets
- A description of any restrictions on data availability
- For clinical datasets or third party data, please ensure that the statement adheres to our policy

scRNA-seq raw and processed data are deposited at GEO under accession number GSE211550.

Research involving human participants, their data, or biological material

Policy information about studies with human participants or human data. See also policy information about sex, gender (identity/presentation), and sexual orientation and race, ethnicity and racism.

Reporting on sex and gender	NA
Reporting on race, ethnicity, or other socially relevant groupings	NA
Population characteristics	NA
Recruitment	NA
Ethics oversight	NA

Note that full information on the approval of the study protocol must also be provided in the manuscript.

Field-specific reporting

Please select the one below that is the best fit for your research. If you are not sure, read the appropriate sections before making your selection.

☒ Life sciences ☐ Behavioural & social sciences ☐ Ecological, evolutionary & environmental sciences

For a reference copy of the document with all sections, see nature.com/documents/nr-reporting-summary-flat.pdf

Life sciences study design

All studies must disclose on these points even when the disclosure is negative.

Sample size	Sample sizes were chosen in accordance to test experiments and previous experiments directed in the lab (Christoffersson et al., 2010, 2012; Vågesjö et al. 2021). This previous knowledge allowed us to estimate how large could be the difference between the experimental groups and how much was the variability of the samples in the various experiments. Then, we choose sample sizes that allowed us to test our hypothesis but minimizing the number of animals according to ethical protocols and guidance.
Data exclusions	Outlier were identified using Grubb's test (alpha=0.05) and excluded only with the co-existent knowledge of sampling problems (eg. animal sickness) or measurement errors.
Replication	In average, experiments were replicated independently for 3 times, with a minimum of 2 (scRNA sequencing) and a maximum of 4 times.
Randomization	Animals were allocated to each group in order to guarantee similar gender and age distribution between experimental groups.
Blinding	Blinding was used when performing Laser Spekle flowmetry and assessment of functional recovery of hindlimb. For experiment of in vivo imaging. Flow cytometry and immunohistochemistry, complete blinding was not possible due to the use of already labelled cells in certain samples. In all experiments, we processed sample and acquired data committing ourself to use unbiased methods even when blinding was not possible.

Reporting for specific materials, systems and methods

We require information from authors about some types of materials, experimental systems and methods used in many studies. Here, indicate whether each material, system or method listed is relevant to your study. If you are not sure if a list item applies to your research, read the appropriate section before selecting a response.

Materials & experimental systems

n/a	Involved in the study	
☐	☒	Antibodies
☒	☐	Eukaryotic cell lines
☒	☐	Palaeontology and archaeology
☐	☒	Animals and other organisms
☒	☐	Clinical data
☒	☐	Dual use research of concern
☒	☐	Plants

Methods

n/a	Involved in the study	
☒	☐	ChIP-seq
☐	☒	Flow cytometry
☒	☐	MRI-based neuroimaging

Antibodies

Antibodies used

All antibodies used are described in Extended Data Table 1.

Validation

All antibody used in this study were tested for the intended methods by the production company. Table 1 in the manuscript has the complete list of antibodies with catalog number and manufacture to read about the tests conducted by the company. In addition, we tested each antibody for dilution and specificity in each of our applications and samples. Antibody concentration were determined based on manufacturers instructions and by titration in each specific sample type in our laboratory. Specificity of staining was determined by comparison with isotype controls (as shown in Fig. 1, 5 and 6, as well as the Extended Data Fig. 1, 2, 5, 7 and 9 when gating strategies are clarified) and using only secondary antibody samples in immunohistochemistry.

Animals and other research organisms

Policy information about studies involving animals; ARRIVE guidelines recommended for reporting animal research, and Sex and Gender in Research

Laboratory animals

C57Bl/6J mice (C57BL/6JBomTac, Taconic Biosciences, Bomholt Danmark), Cx3cr1CreERT2 (B6.129P2©-Cx3cr1tm2.1(cre/ERT2)Jung/J, RRID:IMSR_JAX:020940, Yona et al., 2013), Rosa26tdTomato mice (Ai14, B6.Cg-Gt(ROSA)26Sortm14(CAG-tdTomato)Hze/J, RRID:IMSR_JAX:007914,Madisen et al.2010), PdgfrβeGFP mice (Tg(Pdgfrb-EGFP)JN169Gsat/Mmucd, RRID: MMRRC 031796-UCD, Gong et al., 2003), Cx3cr1GFP/+ mice (B6.129P2(Cg)-Cx3cr1tm1Litt/J, RRID:IMSR_JAX:005582, Jung et al., 2000), Pdgfrβflx/flx, kindly provided by Professor Betsholtz (Uppsala, Sweden) and Ng2dsRed mice (Tg(Cspg4-DsRed.T1)1Akik/J, RRID:IMSR_JAX:008241, Zhu et al., 2008) were used in the current study. All animals used were males and females mixed, from 6 to 15 weeks old. Mice were kept as a rule in groups of 5 per cage, and 2 per cage after major surgery. The plastic cages had a layer of wood shavings covering the floor and contained bedding enrichment. Cages, food, and water bottles were changed twice a week, and supervision was carried out daily.

Wild animals

The study did not involve wild animals.

Reporting on sex

Both male and female mice were included in the study and selected to assure similar distribution between experimental groups.

Field-collected samples

The study did not involve samples collected in the field.

Ethics oversight

All experiments were approved by the Uppsala Region Laboratory Animal Ethics Board (numbers C81/14 and C12740/20).

Note that full information on the approval of the study protocol must also be provided in the manuscript.

Plants

Seed stocks

NA

Novel plant genotypes

NA

Authentication

NA

Flow Cytometry

Plots

Confirm that:

☒ The axis labels state the marker and fluorochrome used (e.g. CD4-FITC).

☒ The axis scales are clearly visible. Include numbers along axes only for bottom left plot of group (a 'group' is an analysis of identical markers).

☒ All plots are contour plots with outliers or pseudocolor plots.

☒ A numerical value for number of cells or percentage (with statistics) is provided.

Methodology

Sample preparation	The mice were euthanized by cervical dislocation, after which the gastrocnemius muscle or the islet graft was removed and minced into very small pieces with a scalpel. The tissue was then mechanically and enzymatically dissociated as follows: the muscle was incubated in collagenase II (500 U/ml) (#17101015; Gibco) in RPMI1640 in 37°C for 30 minutes during intermittent pipetting. After washing the tissue with ice-cold DPBS, it was incubated for further 30 minutes with collagenase IV (15 U/ml) (#17104019; Gibco) and dispase (2.4 U/ml) (#17105041; Gibco) in RPMI1640, with intermittent pipetting. Thereafter, the tissue suspension was passed through a 23G 0.6 mm needle 8-10 times and filtered through a 70 µm cell strainer. For flow cytometry, single-cell suspensions were subjected to debris removal (#130-109-398; Miltenyi Biotec), followed by incubation with 10% FBS in RPMI-1640 for 20 minutes on ice. Next, extracellular antigen fluorochrome-conjugated primary antibodies were added for 15 minutes on ice, with appropriate control antibodies (Table 1). Last, live/dead cell staining was carried out using CellTrace Calcein Violet, AM (C34858; Invitrogen) or LIVE/DEAD Fixable Violet Dead Cell Stain Kit (L34964; Invitrogen), according to manufacturer's instructions.
Instrument	Cells were analyzed using CytoFlex S (Beckman Coulter) flow cytometer with CytExpert software (Beckman Coulter) or Cytek® Northern Lights™ in the 3 laser configuration (16V-14B-8R).
Software	Data analysis was performed using FlowJo software (BD).
Cell population abundance	10.3±1.4 and 13.0±1.5% of macrophages in ischemic muscles express PDGFRβ at 7 and 21 days after HLI.
Gating strategy	Macrophages are gated as singlets/Live cells/CD45+/F4/80+/CX3CR1+, with population of interest (mural cell-like macrophages) also being PDGFRβ+. Additionally, in lineage tracing experiments, mural cell-like macrophages are gated as inglets/Live cells/tdTomato+/eGFP+. Gating strategies are represented in Fig.1E, Fig. 5D, Extended Data Fig.1A, Extended Data Fig.6C, and Extended Data Fig.8B.

☒ Tick this box to confirm that a figure exemplifying the gating strategy is provided in the Supplementary Information.
